# Identification of nitric oxide-mediated necroptosis as the predominant death route in Parkinson’s disease

**DOI:** 10.1186/s43556-024-00213-y

**Published:** 2024-10-24

**Authors:** Ting Zhang, Wenjing Rui, Yue Sun, Yunyun Tian, Qiaoyan Li, Qian Zhang, Yanchun Zhao, Zongzhi Liu, Tiepeng Wang

**Affiliations:** 1 School of Medicine, Shihezi University, Shihezi, 832000 China; 2 Key Laboratory of Xinjiang Endemic and Ethnic Diseases, School of Medicine, Shihezi University, Shihezi, 832000 China; 3 Changping Laboratory, Beijing, 102206 China; 4 Prenatal Diagnosis Center of Urumqi Maternal and Child Health Hospital, Urumuqi, 830000 Xinjiang China; 5 Key Laboratory of Genomic and Precision Medicine, Beijing Institute of Genomics, Chinese Academy of Sciences, Beijing, 100101 China; 6 Key Laboratory of Biomacromolecules (CAS), National Laboratory of Biomacromolecules, CAS Center for Excellence in Biomacromolecules, Institute of Biophysics, Chinese Academy of Sciences, Beijing, 100101 China

**Keywords:** Nitric oxide, Necroptosis, Parkinson's disease, Neurodegenerative disease

## Abstract

Parkinson's disease (PD) involves multiple forms of neuronal cell death, but the dominant pathway involved in disease progression remains unclear. This study employed RNA sequencing (RNA-seq) of brain tissue to explore gene expression patterns across different stages of PD. Using the Scaden deep learning algorithm, we predicted neurocyte subtypes and modelled dynamic interactions for five classic cell death pathways to identify the predominant routes of neuronal death during PD progression. Our cell type-specific analysis revealed an increasing shift towards necroptosis, which was strongly correlated with nitric oxide synthase (NOS) expression across most neuronal subtypes. In vitro experiments confirmed that nitric oxide (NO) is a key mediator of necroptosis, leading to nuclear shrinkage and decreased mitochondrial membrane potential via phosphorylation of the PIP1/PIP3/MLKL signalling cascade. Importantly, specific necroptosis inhibitors significantly mitigated neuronal damage in both in vitro and in vivo PD models. Further analysis revealed that NO-mediated necroptosis is prevalent in early-onset Alzheimer's disease (AD) and amyotrophic lateral sclerosis (ALS) and across multiple brain regions but not in brain tumours. Our findings suggest that NO-mediated necroptosis is a critical pathway in PD and other neurodegenerative disorders, providing potential targets for therapeutic intervention.

**Supplementary Information:**

The online version contains supplementary material available at 10.1186/s43556-024-00213-y.

## Introduction

Neurodegenerative diseases are major challenges in medical science and are characterized by a gradual decline in nervous system function and structure. Common neurodegenerative diseases include Alzheimer's disease (AD), Parkinson's disease (PD), and amyotrophic lateral sclerosis (ALS) [1–3]. These conditions are characterized by various symptoms that progressively impair cognitive and motor functions, substantially impacting the life quality of patients [4, 5]. Current treatment options primarily target symptoms, with limited therapies available to halt or slow the underlying pathological processes [6–9]. Elucidation of the patterns and underlying mechanisms of neuronal cell death has important therapeutic implications.

Recent studies have revealed various mechanisms of cell death involved in neurodegeneration, including apoptosis, autophagy, ferroptosis, necroptosis, and parthanatos [10–12]. While each pathway can independently contribute to neuronal loss, these pathways often interact and influence each other. For example, the interplay between autophagy and apoptosis can be modulated by the second messenger calcium (Ca^2+^), which impacts neuronal excitability [13]. Another example involves caspase-8 acting as a mediator between the apoptotic and necrotic pathways, serving as a pivotal link among diverse cell death mechanisms [14]. Our study focuses on these five classic cell death pathways because they encompass a spectrum of cell death mechanisms, both programmed and unprogrammed. The well-documented molecular mechanisms of these pathways provide a robust foundation for research and potential therapeutic targets. Additionally, these pathways are integral to the pathology of neurodegenerative diseases, with apoptosis and autophagy involved in clearing damaged components and necroptosis and ferroptosis linked to inflammation and membrane damage. Previous research and experiments with inhibitors of these pathways have further highlighted their therapeutic potential. Investigating the transition and interaction between these death modes throughout the progression of neurodegenerative diseases can offer valuable insights into the development of these conditions. A deeper understanding of these interactions may facilitate the identification of effective intervention strategies and therapeutic targets for neurodegeneration [15, 16].

Nitric oxide (NO) has been implicated in the pathogenesis of neurodegenerative disorders, including PD, where it is suggested to mediate disease progression [17, 18]. Our preliminary investigations revealed that the NO donor GSNO can induce necroptosis-like damage in neuronal cells in vitro; necroptosis is an important type of neuronal cell death in degenerative diseases. Hence, NO may mediate neuronal damage through the induction of necroptosis [19].

In this study, we employ a multitiered research approach to delineate the primary pathways of neuronal cell death patterns during the progression of degenerative disease and investigate the role and mechanism of NO in mediating related illnesses, offering a scientific foundation and practical insights for the prevention and treatment of neurodegenerative conditions.

## Results

### Expression characteristics of PD patients at different pathological stages

**Fig. 1:**
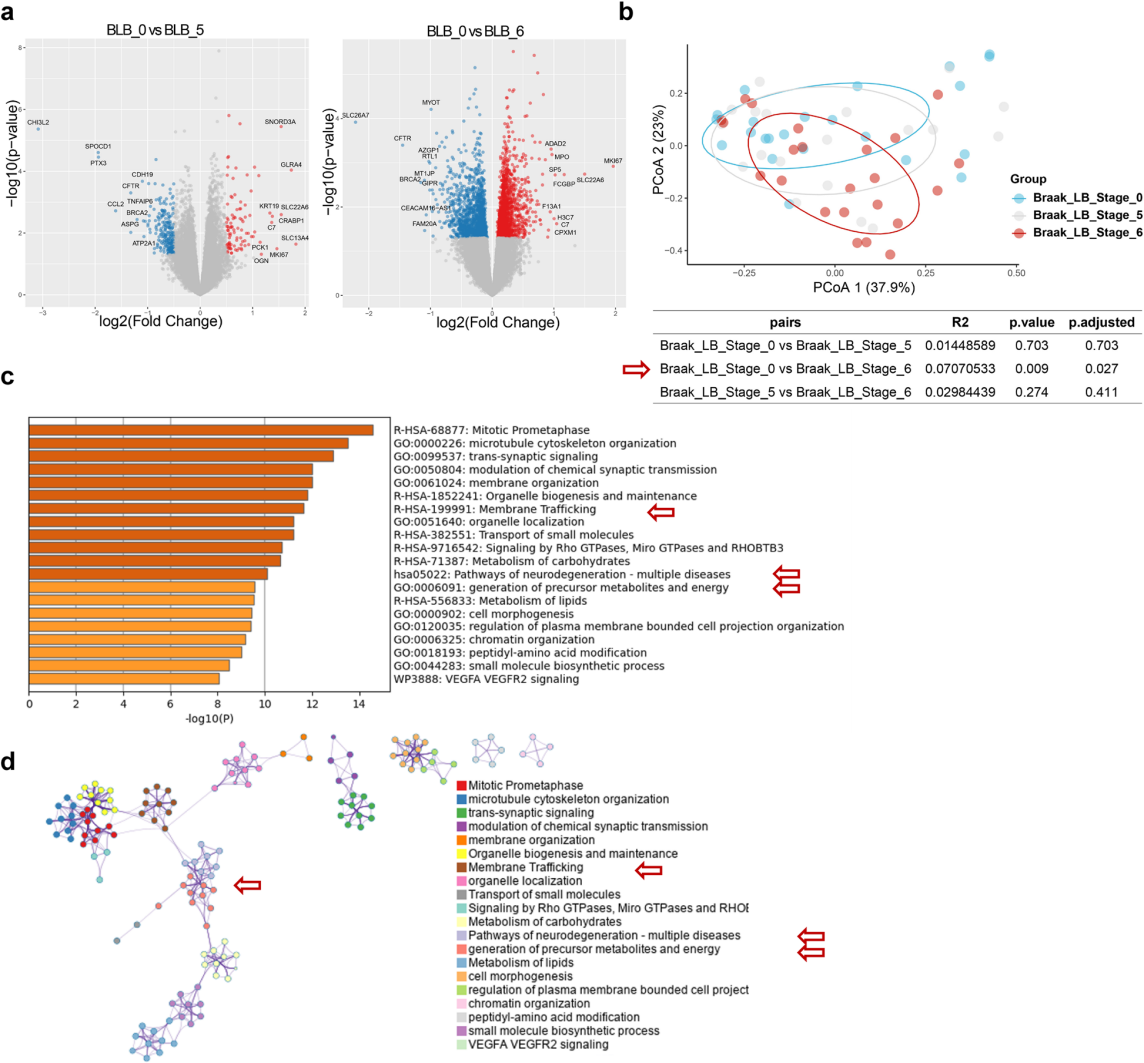
Genomic expression profile of PD at different pathological stages. **a** DEGs at different pathological stages. (Blue dots represent significant downregulation, red dots represent significant upregulation, |log2FC|≥ 1, *P* ≤ 0.05). **b** PCoA at different pathological stages (there was a statistically significant difference in the gene expression profiles of brain tissues at BLB6 and BLB0, *P* value < 0.05). **c** Pathway enrichment of DEGs between BLB6 and BLB0; the DEGs are enriched predominantly in various pathways associated with the onset of neurodegenerative diseases (indicated by the red arrow). **d** Pathway interaction network between BLB6 and BLB0. Pathways of neurodegeneration are at the core of the interactive network, indicated by the red arrow, each term is represented by a circle node, where its size is proportional to the number of input genes fall under that term, and its color represent its cluster identity (i.e., nodes of the same color belong to the same cluster). Terms with a similarity score > 0.3 are linked by an edge (the thickness of the edge represents the similarity score). The network is visualized with Cytoscape with “force-directed” layout and with edge bundled for clarity

Neurodegenerative diseases are characterized by the progressive loss of neuronal cells through complex mechanisms [20]. First, we aimed to delineate the dynamic transcriptomic landscape during the progression of neurodegenerative diseases. We analysed brain tissue samples from patients with PD stratified according to the BLB stage. Comparative analysis (Fig. 1a and Fig. S1) revealed significant differences in gene expression profiles among BLB0, BLB5 and BLB6 (Fig. 1a). With increasing disease severity, the number of differentially expressed genes (DEGs) increased from 2,611 to 2,691. Notably, the upregulated genes exhibited the most pronounced changes, with 1,327 and 1,243 genes upregulated in BLB6 and BLB5, respectively (Fig. S1a). As the disease progressed, we observed an increase in the Euclidean distance of the DEGs of the patients, as shown by principal coordinate analysis (PCoA) in Fig. 1b. Specifically, patients at BLB5 presented intermediate distances between stages 0 and 6, indicating dynamic and disease severity-dependent progression in PD.

To elucidate the molecular mechanisms underlying disease progression, we subsequently performed pathway annotation (Fig. 1 c, Fig. S1b) and interaction network analysis (Fig. 1d, Fig. S1c). The DEGs were enriched predominantly in pathways such as 'Pathways of Neurodegeneration—Multiple Diseases', 'Generation of Precursor Metabolites and Energy' and 'Membrane Trafficking' (Fig. 1c). The enrichment of DEGs within the 'Pathways of Neurodegeneration—Multiple Diseases' pathway indicates a potential role for these genes as key drivers in the aetiology and progression of various neurodegenerative conditions. Intriguingly, 'Generation of Precursor Metabolites and Energy' and 'Membrane Trafficking' were found at the core of the gene regulatory network (Fig. 1d, Table S1), which indicates that abnormalities in energy metabolism may play critical roles in neuronal cell death during PD progression. To test this hypothesis, we examined creatine kinase family genes, which are core genes related to cell energy storage and buffering [21, 22]. We observed significant downregulation of CKMT1A and CKMT1B in brain tissues at BLB6 compared with BLB5, as shown in Fig. S1D. Given the pivotal roles of these molecules in energy metabolism, these results suggest that deficits in energy storage may strongly contribute to neuronal cell death and the progression of PD.

### Necroptosis is the predominant death route in PD progression

**Fig. 2:**
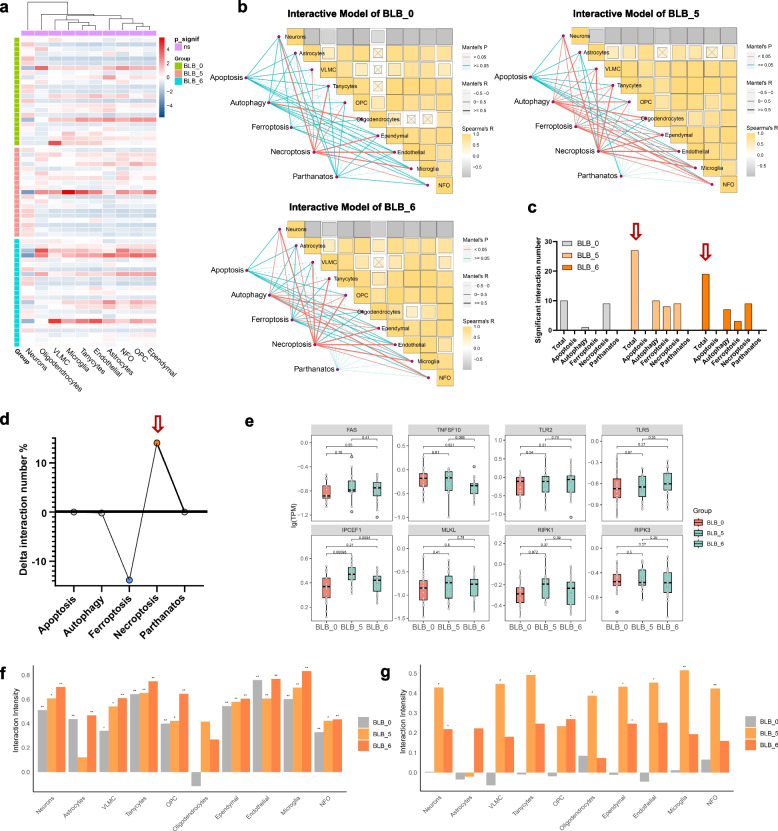
Dynamic model of the neurocyte death route during the progression of PD. **a** Neurocyte subtype prediction via deconvolution algorithms (the heatmap shows the proportion of neural cells; purple indicates no significant difference, *P* > 0.05). **b** Dynamic interaction model between neurocytes and five classic death patterns. (The left side represents the mode of death, the right side represents the type of neurocyte, and the lines represent interaction effects. The colour of the line represents significance, where red represents *P* < 0.05, and green represents *P* > 0.05. The thickness of the line represents the strength of the interaction. The correlation heatmap on the right represents the interaction between the death pathway and neurocytes. The colour represents the Spearman correlation coefficient.). **c** Statistics on significant interaction numbers during the progression of PD. (As the disease progresses, the total number of interactions increases, indicated by the red arrow). **d** Statistics on significant changes in interaction numbers during the progression of PD. (As the disease progresses, necroptosis increases while ferroptosis decreases, blue dots represent decreases, while red dots represent increases, indicated by the red arrow). **e** Expression of marker genes for necroptosis during the progression of PD (the x-axis represents different periods, the y-axis represents lgTPM, and *P* < 0.05 indicates a statistically significant difference). **f** Analysis of necroptosis correlation strength across different neural cell types at varying BLB grading levels. The results revealed a notable increase in necroptosis correlation strength as BLB grading progressed across nearly all neural cell types, including neurons, astrocytes, VLMCs, tanycytes, OPCs, oligodendrocytes, ependymal cells, endothelial cells, microglia, and NFOs. Specifically, in astrocytes and endothelial cells, the correlation strength decreases at BLB5 but increases again at BLB6. Conversely, oligodendrocytes show the highest correlation strength at BLB5, which decreases at BLB6 but remains higher than at BLB0. **g** Analysis of ferroptosis correlation strength across the same neural cell types during the transition from BLB5 to BLB6. A decrease in ferroptosis correlation strength is observed across all cell types, supporting the hypothesis that as neurodegenerative diseases progress, there is a shift towards necroptosis with increasing disease severity. The pattern of cell death mechanism diversity is evident across the neural cell types analysed

Our objective was to identify the predominant cell death mechanisms associated with the progression of PD. By employing deep learning algorithms, we classified neural cell types within brain tissues and developed dynamic interaction models for five classic modes of cell death: apoptosis, autophagy, ferroptosis, necroptosis and parthanatos. While we observed no significant disparities in neural cell subtypes between the healthy controls and the PD patients (Fig. 2a), two notable trends emerged. First, with advancing PD, diverse modes of cell death were observed in brain tissues (Fig. 2b, c). Second, an increase in disease severity coincided with a pronounced shift towards necroptosis, marked by an increased prevalence of this pathway and a concurrent decline in autophagy and ferroptosis (Fig. 2b, d). These observations indicate that necroptosis may be a predominant regulatory mechanism in PD pathogenesis. Furthermore, genes pivotal to necroptosis, including FAS, TLR2, and TLR5, were upregulated in advanced BLB6 relative to BLB5 (Fig. 2e). Conversely, genes linked to apoptosis and autophagy were downregulated (Fig. S2), confirming that the transition towards necroptosis is a critical pathway in the progression of PD.

Furthermore, we aimed to determine the cell type-specific characteristics of necroptosis. Overall, our observations indicate that with the progression of BLB grading, there is a notable increase in the correlation strength of necroptosis across nearly all neural cell types, including neurons, astrocytes, vascular leptomeningeal cells (VLMCs), tanycytes, oligodendrocyte progenitor cells (OPCs), oligodendrocytes, ependymal cells, endothelial cells, microglia, and newly formed oligodendrocytes (NFOs). Specifically, variations among cell types revealed that, in astrocytes and endothelial cells, the correlation strength decreases at BLB5 but subsequently increases at BLB6. Conversely, oligodendrocytes exhibited the highest correlation strength at BLB5, which then diminished at BLB6, although it remained elevated compared with that at BLB0 **(**Fig. 2f**)**. Furthermore, our findings revealed a decrease in the degree of ferroptosis correlation across all cell types during the transition from BLB5 to BLB6 **(**Fig. 2g**)**. These results support our hypothesis that as neurodegenerative diseases progress, the diversity of cell death mechanisms broadens, and with increased disease severity, there is a preferential shift towards necroptosis. This pattern was evident across almost all analysed neural cell types.

### NOS is involved in the regulation of necroptosis in PD, but it does not have a role in brain tumours

**Fig. 3:**
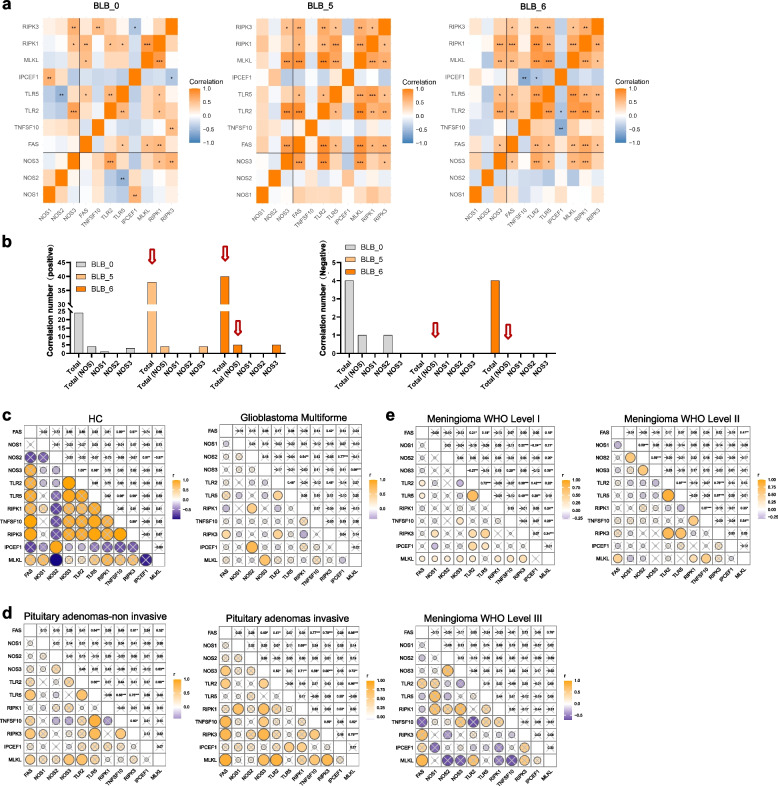
Association analysis of NOS with key genes involved in necroptosis. **a** Correlation heatmap showing the associations between NOS and key genes involved in necroptosis. Orange represents a positive correlation, and blue represents a negative correlation. (*, *P* < 0.05; **, *P* < 0.01; ***, *P* < 0.001). **b** Statistics of significantly positively and negatively correlated numbers. (As the disease progresses, the positive association between NO and necroptosis increases, while the negative association decreases, indicated by the red arrow). **c** In glioblastoma, the correlation between NO-mediated necroptosis and tumour progression significantly decreases as the tumour develops. **d** In pituitary adenomas, the correlation between NO-mediated necroptosis is more common in invasive tumours than in non-invasive tumours. **e** In meningiomas, the correlation strength of NO-mediated necroptosis gradually decreases with increasing WHO grade, indicating a reduced association in more aggressive forms of the tumour

Our research revealed that GSNO, a donor of NO, induced a form of neural cell death resembling necroptosis, marked by disrupted cell membranes observable 5–6 h post-treatment rather than immediately after the stimulus (Fig. S3). These findings indicated that NO may mediate neuronal necroptosis in the pathogenesis of PD. To validate this finding, we initially assessed the relationship between NOS family genes and those pivotal in necroptosis. We observed that with PD progression, the correlation between NOS genes and necroptotic genes, particularly NOS3, significantly increased. Moreover, the correlation between NOS1 and necroptotic genes generally increased, although the difference did not reach statistical significance (Fig. 3a). Furthermore, statistical analysis of the interactions between the NOS family and necroptotic genes revealed a significant increase in positive interactions and a corresponding decrease in negative interactions with PD progression (Fig. 3b). These results suggest that NO-mediated necroptotic cell death could be a central mechanism in the progression of PD.

Moreover, we conducted a comparative analysis using human brain tumours, as these diseases also exhibit chronic progression and are related to the nervous system. We found that NO-mediated necroptosis is not conserved in brain tumours and that most of these processes are not significant. In glioblastoma, the correlation between NO-mediated necroptosis significantly decreased following tumour development (Fig. 3c). In pituitary adenomas, the correlation was greater in invasive tumours than in noninvasive tumours (Fig. 3d). In meningiomas, this correlation gradually diminished with increasing WHO grade (Fig. 3e). These findings provide counterevidence that NO-mediated necroptosis is universally prevalent in classic neurodegenerative diseases but may not be conserved in other brain diseases.

### NO-mediated necroptosis is the central mechanism of neural cell death

We identified NO-mediated necroptosis as the principal mechanism underlying neural cell death in PD. To substantiate our findings through computational analysis, we developed an in vitro PD model in which the neural cell line SH-SY5Y was exposed to rotenone to establish that NO-induced necroptosis is a central pathway for cell death.

**Fig. 4:**
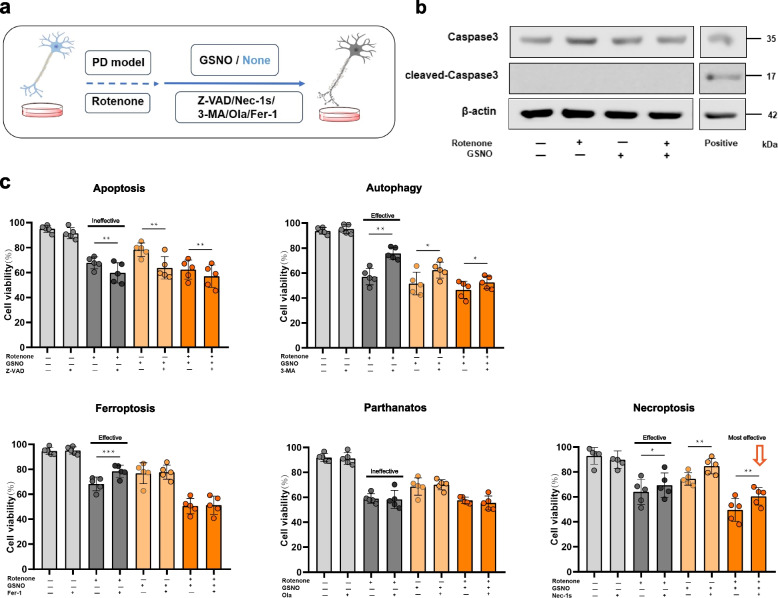
NO-mediated necroptosis in a PD cell model. **a** Experimental workflow: The neural cell line SH-SY5Y was treated with rotenone, with or without the NO donor GSNO, to establish a cellular model for PD. Specific inhibitors targeting various cell death pathways were utilized to identify the pathways involved in the death of the cell line. **b** Western blot analysis of the cleavage of the apoptotic marker protein caspase3. **c** NO-induced cell death pathways were distinguished via the use of specific inhibitors targeting different cell death pathways, and the inhibitor of necroptosis has the strongest effect, indicated by the red arrow. (*, *P* < 0.05; **, *P* < 0.01; ***, *P* < 0.001)

Initially, we utilized various inhibitors—Z-VAD for apoptosis, 3-MA for autophagy, Fer-1 for ferroptosis, Ola for parthanatos, and Nec-1s for necroptosis—to delineate the predominant cell death pathway. Additionally, we introduced GSNO, a NO donor, to validate the role of NO in cellular injury (Fig. 4a). Our observations revealed a marked decrease in cell viability in the rotenone-induced PD model (Fig. 4). Notably, the application of Nec-1s, 3-MA, and Fer-1, but not Ola, significantly restored cell viability, suggesting that the cell death mechanisms involved predominantly autophagy, ferroptosis, and necroptosis rather than parthanatos. The apoptotic inhibitor Z-VAD did not affect cell viability, and the ineffective cleavage of caspase 3 (Fig. 4b), an apoptotic marker, indicated that apoptosis is likely not the primary route of cell death in PD pathogenesis.

These findings are congruent with our bioinformatics-derived cell death models, which revealed increased interactions between neurocytes and pathways of autophagy, ferroptosis, and necroptosis during the BLB0 to BLB5 and BLB6 progression stages, with no interaction observed for apoptosis and parthanatos (Fig. 2b). Furthermore, we discovered that NO not only directly induces neurocyte damage but also strongly amplifies the neurotoxic effects of rotenone, indicating a role for NO in mediating neural cell death in PD. Among the inhibitors tested, Nec-1s demonstrated the most efficacious rescue of cell viability (Fig. 4c), suggesting that NO-driven necroptosis is the predominant cell death mechanism in the progression of PD models. Collectively, our in vitro experiments corroborate the bioinformatics model, reinforcing the notion that NO-mediated necroptosis is a central mechanism in neural cell death associated with PD.

### Abnormal chromatin condensation and MMP collapse in neural cells triggered by NO-mediated necroptosis

Specifically, a reduction in the MMP and the condensation of nuclear DNA are characteristic indicators of neuronal degeneration in PD, whereas increased cell membrane permeability is a defining feature of necroptosis. Our subsequent investigations focused on these features of cellular damage within the context of NO-induced necroptotic pathways.

**Fig. 5:**
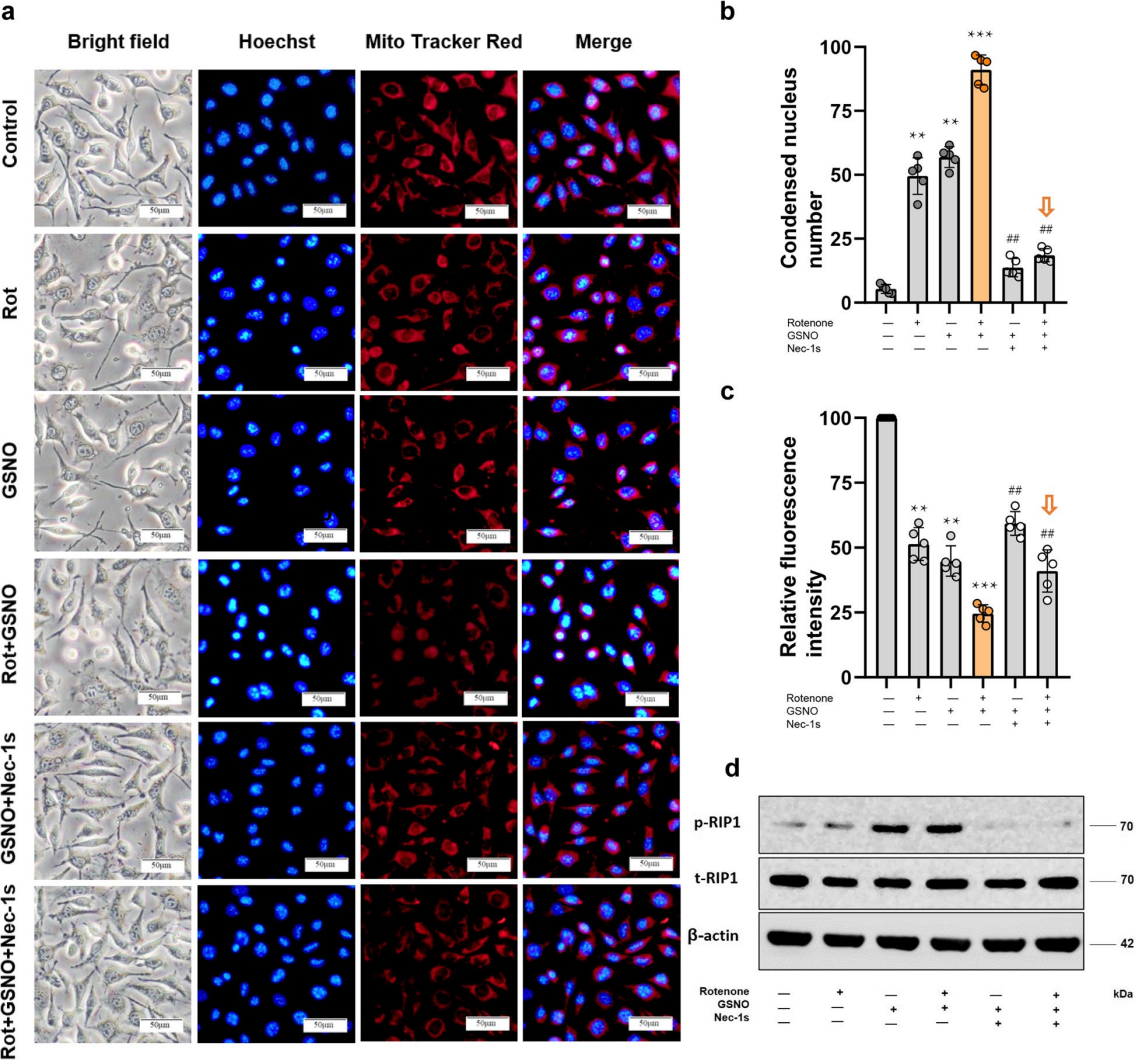
Modulation of nuclear morphology and the MMP by NO-mediated necroptosis. **a** NO-mediated necroptosis triggered nuclear condensation and a decrease in the MMP (blue represents nuclear staining, while red represents the MMP, bar = 50μm). **b** Statistics of nuclear condensation in the neural cells. Nuclei were stained with Hoechst 33342, and the morphology of the nuclei was statistically analysed by randomly selecting ten microscopic fields of view (*, compared with the control group; **, *P* < 0.01; ***, *P* < 0.001. #, compared with the rotenone + GSNO- group; ##, *P* < 0.01). **c** Statistics of the MMP of neural cells. The MMP was characterized by staining with MitoTracker™ Red CMXRos dye, and the relative fluorescence intensity indicated the strength of the MMP. **d** Phosphorylation detection of the necroptotic marker RIP1 via western blotting

We observed notable aberrations in the chromatin structure of neural cells in a rotenone-induced PD model characterized by pronounced DNA condensation and nuclear shrinkage. The introduction of NO exacerbated these chromatin structural anomalies, which were accompanied by cellular oedema and elevated staining with Trypan blue (Fig. 5a, b, Fig. S3). Utilization of Nec-1s, an inhibitor of necroptosis, significantly reduced the proportion of neural cells exhibiting nuclear damage and compromised cell membrane integrity (Fig. 5b, Fig. S3). Concurrently, we noted substantial alterations in the MMP of the affected neural cells. While the MMP was initially reduced in the rotenone-induced PD model, NO resulted in a more pronounced reduction in the MMP. Importantly, Nec-1s significantly attenuated this decrease in the MMP (Fig. 5c).

Moreover, we observed a significant increase in the phosphorylation of receptor-interacting protein kinase 1 (RIP1), a recognized marker of necroptotic cell death. Notably, a marked increase in phosphorylation was observed exclusively following supplementation with NO, and this increase was significantly abrogated in the presence of the necroptosis inhibitor Nec-1s. These findings indicate that NO plays a mediating role in the induction of necroptosis (Fig. 5d).

### Validation of NO-mediated necroptosis in primary neurons

**Fig. 6:**
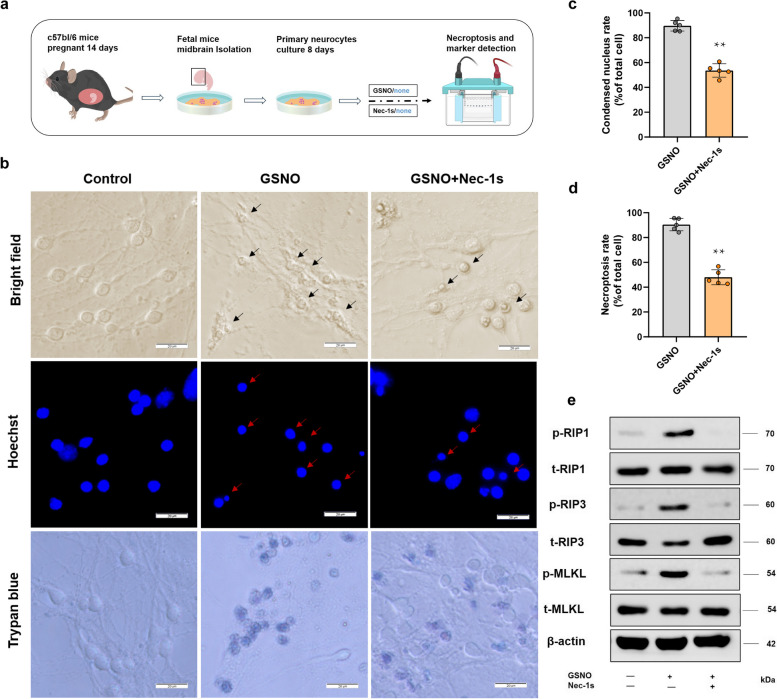
Validation of NO-mediated necroptosis in mouse primary neurons. **a** Experimental workflow: Primary mouse midbrain neurons were cultured and utilized for experimentation, in which the cells were treated with GSNO, either in the presence or absence of the necroptosis-specific inhibitor Nec-1s. The role of necroptosis in primary neurons was confirmed through methodologies including cellular morphological assessment and western blot analysis. **b** Necroptosis in GSNO-treated neurons was detected. Microscopic images of cells under visible light and the distribution of DNA, as revealed by Hoechst 33342 staining, revealed morphological changes in the cell body and nucleus following GSNO treatment. The cells stained with Trypan blue exhibited compromised plasma membrane integrity, bar = 20μm. **c** Statistics of the nuclear condensation rate. Following a 12-h exposure to GSNO, the cells were stained with Hoechst 33342. A total of ten random fields of view were assessed for quantification, and the proportion of condensed nuclei was determined relative to the total cell count within each field. (*, compared with the GSNO group; **, *P* < 0.01). **d** Statistics of the necroptosis rate. One hour following stimulation with GSNO, the cells were rapidly stained with Trypan blue and photographed under a microscope. The proportion of stained cells, indicative of necroptotic injury, was compared with that of the entire group of cells (*, compared with the GSNO group; **, *P* < 0.01). **e** Detection of the phosphorylation cascade of the PIP1/PIP3/MLKL signalling pathway

Primary neurons are considered more representative of the physiological state of nerve cells, as they retain a greater extent of their original biological properties [23, 24]. Therefore, we aimed to further validate the occurrence of NO-induced necroptosis in these cells. Initially, we isolated foetal mouse midbrain neurons (Fig. 6a) and subjected them to NO stimulation and inhibitor treatment. Our findings confirmed that NO-mediated necroptosis is replicable in primary neuronal cultures. Upon NO stimulation, there was a significant increase in the incidence of nuclear shrinkage and a marked increase in cell death, and importantly, the necroptosis inhibitor Nec-1s effectively reversed this deleterious process (Fig. 6b, c, d).

We subsequently aimed to elucidate the molecular mechanisms of NO-mediated necroptosis, with a particular focus on phosphorylation events within the RIP1/RIP3/MLKL cascade, which is known to be a critical juncture in the execution of necroptotic cell death [25, 26]. Our results from primary neuron cultures demonstrated that NO-induced necroptosis is facilitated through the activation of the RIP1/RIP3/MLKL cascade. The initiation of necroptosis is NO dependent, as evidenced by the fact that the phosphorylation cascade occurs only after the addition of the NO donor GSNO (Fig. 6e). On the basis of these findings, we propose that NO-mediated necroptosis is executed via activation of the RIP1/RIP3/MLKL phosphorylation cascade.

### Nec-1s administration mitigates MPTP-induced motor dysfunction and neurodegeneration

**Fig. 7:**
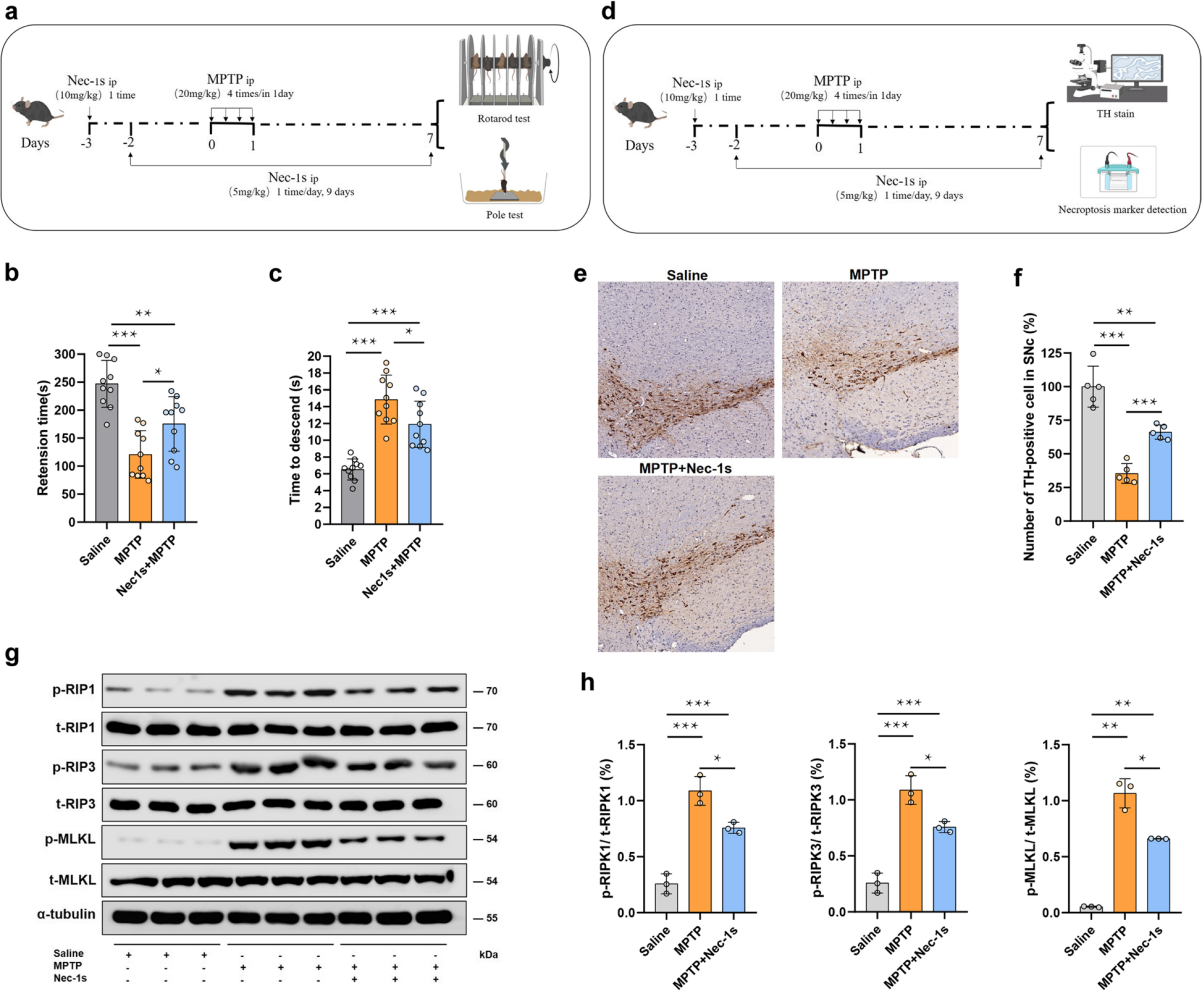
Nec-1s alleviates MPTP-induced motor dysfunction and neurodegeneration in a mouse model of PD. **a** Schematic diagram showing the experimental design. The mice were treated with MPTP (20 mg/kg, i.p.) or saline, followed by Nec-1s (10 mg/kg, i.p.) administration. Behavioural tests (rotarod and pole tests) were conducted to assess motor function. **b** Rotarod test results indicated that MPTP significantly reduced retention time compared with the saline control, whereas Nec-1s treatment partially restored motor function. **c** Pole test results showing that MPTP treatment increased the time taken to descend, which was reduced by Nec-1s administration. **d** Schematic diagram of the experimental design for histological analysis. The mice were treated with MPTP (20 mg/kg, i.p.) or saline, followed by Nec-1s (10 mg/kg, i.p.) administration, followed by immunohistochemical staining and detection of necroptosis markers. **e** Representative images of TH staining in the substantia nigra showing significant loss of TH-positive neurons in the MPTP-treated mice, which was alleviated by Nec-1s treatment (20x). **f** Quantification of TH-positive cells revealed that Nec-1s significantly preserved dopaminergic neurons compared with MPTP treatment alone. **g** Western blot analysis of necroptosis markers, including p-RIP1, p-RIP3, and p-MLKL. MPTP treatment increased the phosphorylation levels of these markers, indicating activation of the necroptosis pathway, which was mitigated by Nec-1s. **h** Densitometric analysis of western blots showing the relative levels of p-RIP1/t-RIP1, p-RIP3/t-RIP3, and p-MLKL/t-MLKL. Nec-1s significantly reduced the phosphorylation of these necroptosis markers in MPTP-treated mice. The data are presented as the means ± SEMs. Statistical significance: *P < 0.05, **P < 0.01, ***P < 0.001

To investigate the neuroprotective effects of Nec-1s against MPTP-induced parkinsonism, we conducted behavioural assessments and analysed neurodegeneration markers in the substantia nigra. In the rotarod test (Fig. 7a, b), the MPTP-treated mice presented a significant reduction in retention time compared with the saline-treated mice, indicating motor dysfunction. However, Nec-1s treatment significantly improved retention times in the MPTP-treated mice, suggesting partial recovery of motor function. Similarly, in the pole test (Fig. 7a, c), the MPTP-treated mice took significantly longer to descend than the control mice did. Nec-1s administration significantly reduced the descent time in the MPTP-treated mice, further supporting the protective effect of Nec-1s on motor coordination. Histological analysis (Fig. 7d, e) revealed that MPTP treatment resulted in a marked decrease in TH-positive neurons in the substantia nigra, indicative of dopaminergic neuron loss. Nec-1s treatment significantly preserved the number of TH-positive cells in the MPTP-treated mice, demonstrating its neuroprotective effect. Western blot analysis (Fig. 7g, h) of necroptosis markers revealed that MPTP treatment increased the phosphorylation levels of RIP1, RIP3, and MLKL, key proteins involved in the necroptosis pathway. Treatment with Nec-1s significantly reduced the phosphorylation of these proteins, indicating that Nec-1s inhibits MPTP-induced necroptosis.

### The universality of NO-mediated necroptosis in AD, PD, and ALS patients and in multiple brain regions

**Fig. 8:**
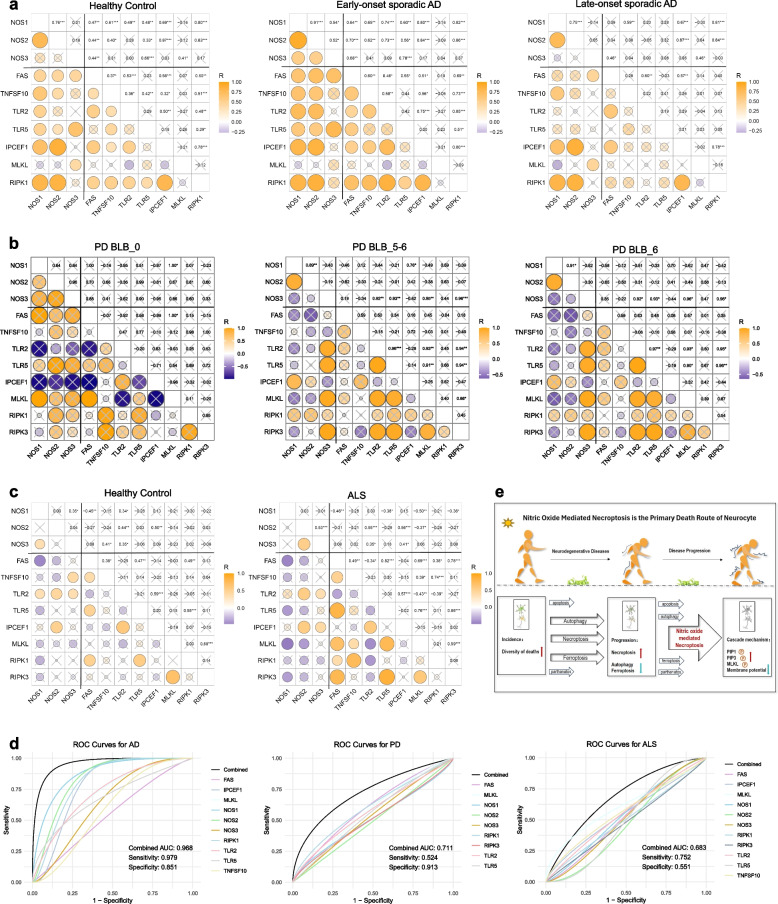
Universality of NO-mediated necroptosis in AD, PD and ALS. **a** Association analysis of NOS with key necroptotic genes in both early-onset and late-onset sporadic AD. **b** Association analysis of NOS with key necroptotic genes in the PD verification cohort. **c** Association analysis of NOS with key necroptotic genes in ALS. Orange represents a positive correlation, and blue represents a negative correlation. *, *P* < 0.05; **, *P* < 0.01; ***, *P* < 0.001; × represents not statistically significant. **d** ROC curve analysis of necroptosis markers as potential biomarkers for disease stratification in PD, AD, and ALS patients. **e** NO-mediated necroptosis is the primary death route of neurocytes. Our research indicates that as neurodegenerative diseases progress, the diversity of cell death patterns initially increases. In the final stages, necroptosis becomes the predominant pathway, a process that is accompanied by the phosphorylation of the PIP1/PIP3/MLKL cascade signalling pathway

Next, we sought to ascertain the universality of NO-mediated necroptosis across various neurodegenerative conditions, with the goal of informing clinical applications. We conducted a comprehensive analysis of patient-derived samples stratified by disease onset and type, encompassing early-onset and late-onset sporadic AD, as well as ALS, and corresponding control samples from a diverse array of brain regions. We also included a new set of PD brain tissue validation samples to verify the reliability of the results. We investigated the correlation between the NOS gene family and pivotal genes implicated in necroptosis, assessing the uniformity of this association across a spectrum of brain tissues. Our findings revealed the universal nature of NO-mediated necroptosis. In the context of AD, patients with early-onset sporadic AD presented a greater positive correlation between NOS expression and markers of necroptosis. In contrast, patients with late-onset sporadic AD presented a diminished correlation, suggesting a potential link between NO-mediated necroptosis and disease progression. (Fig. 8a). In the PD validation samples, we likewise observed an increase in association as the disease progressed (Fig. 8b). In the individuals with ALS, the correlation between NO-induced necroptosis and disease onset was significantly greater than that in the healthy controls. Additionally, we conducted an in-depth analysis to determine the relationship between necroptosis and various cell types within distinct brain regions, including the frontal cortex and motor cortex. This evaluation included neurons, oligodendrocyte lineage cells, and other glial cells. Our findings revealed a pronounced correlation, suggesting a potential role of NO-mediated necroptosis in the cellular pathology of ALS. Although some of the associations were not statistically significant, we still found trends indicative of a correlation, such as an increase in the number of correlations and a rise in the magnitude of the correlation coefficients (Figs. 8c, S4).

Collectively, these observations indicate that NO-mediated necroptosis is a fundamental mechanism underlying the aetiology and progression of classic neurodegenerative diseases, including AD, PD, and ALS.

Finally, we conducted multifactorial logistic regression analyses to assess the efficacy of necroptosis markers as potential biomarkers for disease stratification in PD, AD, and ALS patients via ROC curves (Fig. 8d). In the context of PD, the combined application of necroptosis markers indicates a moderate classification capacity and presents a low sensitivity and high specificity, suggesting that this method is highly effective at identifying negative samples but less adept at detecting positive samples. These results indicate a need for further optimization to increase the sensitivity and overall classification capability of this method. In contrast, for AD, necroptosis markers demonstrate robust classification performance, positioning them as excellent biomarkers for disease stratification with high sensitivity and specificity, reflecting their strong ability to distinguish various disease states. Conversely, in ALS, although the classification capacity of these markers is detectable, it is comparatively less pronounced than their utility in AD and PD.

## Discussion

Our study revealed three important findings: 1) We revealed the diversity of neuronal cell death mechanisms, particularly highlighting necroptosis as the primary pathway involved in the progression of neurodegenerative diseases. 2) We validated the critical role of NO in mediating necrotic apoptosis. This mechanism involves the PIP1/PIP3/MKLK phosphorylation cascade (Fig. 8e). 3) We confirmed the universality of NO-mediated necroptosis across various types of classic neurodegenerative diseases and multiple brain regions.

Currently, research on the mechanisms of neuronal cell death encompasses multiple aspects, such as apoptosis, autophagy, necroptosis, and ferroptosis, which are crucial for the development of neurodegenerative diseases [10, 20, 27, 28]. However, only by clearly delineating the macroscopic spectrum of neuronal cell death patterns can we comprehensively understand the temporal and spatial characteristics of disease progression [29].

Our findings indicate that during the progression of neurodegenerative diseases, neural cells exhibit a convergent death pattern—necroptosis—regulated by NO. As BLB grades increase, necroptosis becomes more pronounced across all neural cell types. Astrocytes and endothelial cells initially show a temporary reduction in necroptosis at BLB5 but increase at BLB6. Oligodendrocytes peak in necroptosis at BLB5, with a decline at BLB6 still above baseline. In contrast, ferroptosis decreases across all cell types from BLB5 to BLB6.

Based on these findings, we speculate in the early stages, cells may primarily clear damaged components through cell death mechanisms, such as apoptosis and autophagy, which help maintain neuronal function. However, as the disease progresses and the pathological environment deteriorates, cell death pathways such as necroptosis and ferroptosis gradually become dominant. This shift may be closely related to factors such as neuroinflammation and oxidative/nitrosative stress, with a significant increase likely due to their association with inflammatory responses and cellular membrane damage. Although our experiments cannot explain why cells utilize this strategy, it may represent a characteristic feature of exacerbated neurodegenerative diseases. Alternatively, this convergent death pattern may serve as a compensatory self-rescue process for the organism, as studies suggest that glial cells are sensitive to iron death, that reversing iron death can reduce glial cell death [30], and that microglial ferroptosis is regulated by SEC24B and contributes to neurodegeneration [31]. Therefore, cells may actively select death modes to avoid further exacerbation of self-injury [32–34]. However, constrained by changes in the brain microenvironment and organismal ageing, this compensatory death mode adjustment may not reverse the progression of the disease [35–37].

Precise modulation of NO can ameliorate oxidative stress status and signal transduction in neural cells, thereby alleviating neuronal degeneration and synaptic loss during the progression of relative diseases [38, 39], and have good value in the formulation of clinical treatment strategies [40]. We have performed both cellular and mouse experiments and confirmed that NO is an important executor of necroptosis. Based on our results, this process is initiated by the phosphorylation of the PIP1/PIP3/MLKL signalling cascade, which underscores the intricate molecular mechanisms of NO-mediated necroptosis.

Neuroinflammation is a key feature of PD, where inflammatory cytokines like TNF-α and IL-1β activate the RIPK1/RIPK3 complex, enhancing the phosphorylation of the PIP1/PIP3/MLKL cascade. This activation triggers MLKL, leading to plasma membrane destruction and further inflammation, creating a cycle of damage. Mitochondrial dysfunction and oxidative stress, common in PD, may also affect PIP1/PIP3 through pathways like AMPK and mTOR, driving necroptotic neuronal death and worsening neuroinflammation as the disease progresses [41–43]. The development of specific inhibitors targeting the RIP1/RIP3/MLKL pathway holds promise for delaying the process of necroptosis and associated diseases. Current necroptosis inhibitor development primarily focuses on RIP1, with some targeting RIP3 or MLKL [43]. Several necroptosis inhibitors, including GSK2982772 and GSK3145095, have entered clinical trials but face issues like off-target effects, metabolic instability, and limited efficacy, with GSK2982772 failed in trials and caused severe rheumatoid arthritis [43]. Future development should focus on enhancing efficacy while minimizing side effects and improving drug stability and administration.

Lastly, our study demonstrates the widespread involvement of NO-mediated necroptosis in the pathogenesis of PD, AD, and ALS, with a consistent trend observed across various brain regions. ROC analysis further confirmed that necroptosis-related molecules could serve as potential biomarkers for predicting disease progression, offering new avenues for early diagnosis and personalized treatment. The development of blood-based biomarkers to reflect tissue conditions may represent a promising direction for early diagnosis in the future.

Our study has limitations, including insufficient samples from BLB1 to BLB4 levels to fully capture early neurodegenerative patterns in PD. Additionally, while we propose and validate the role of NO-mediated necroptosis, further investigation into its detailed molecular and cellular mechanisms is needed for a more comprehensive understanding of neuronal cell death regulation.

In summary, our study reveals the universality and mechanism of NO-mediated necroptosis in neurodegenerative diseases. Targeting the NO-mediated necroptosis pathway may offer promising avenues for the development of novel treatments to combat neurodegenerative diseases.

## Methods and materials

### Data source and preprocessing

The present study was based on public data from the Gene Expression Omnibus (GEO) database [44, 45]. We conducted a keyword search using AD, PD, and ALS, ultimately identifying three viable brain tissue cohorts for our study. These datasets were GSE216281, featuring PD samples with Braak Lewy body (BLB) stages of 0–6 (*n* = 84); GSE203206, comprising early-onset sporadic AD, late-onset sporadic AD, and control samples (*n* = 47); and GSE219278, encompassing ALS and control samples from multiple brain regions (*n* = 84). We also used GSE168496 as a validation set for PD (*n* = 16), the glioblastoma multiforme dataset GSE263588 (*n* = 49), the meningioma dataset GSE252291 (*n* = 279), and the pituitary adenoma dataset GSE260487 (*n* = 32) as reverse validation sets. Upon integrating the data, we noted that the GSE216281 cohort had a limited number of samples in BLB stages 1–4, with the lowest representation being only two sample. This lack of samples posed a potential risk of reduced statistical power; therefore, we removed these data while retaining all the samples from the other datasets.

### Differential expression gene analysis and pathway annotation

We removed low-quality counts and lncRNAs, and the counts were then converted to transcripts per million (TPM) values for subsequent analyses. Differential gene expression analysis was conducted via the EdgeR package in R [46], with the criteria for significance set at |log2FoldChange (FC)|≥ 1 and *P* ≤ 0.05. Volcano plots were generated to visualize these differences. The significant genes were subsequently subjected to pathway enrichment analysis and gene interaction network construction via Metascape [47], providing comprehensive insight into the molecular mechanisms underlying the observed gene expression changes.

### Neural cell subtype prediction through the scaden algorithm

The inherent challenge in using bulk-tissue RNA sequencing (RNA-seq) is its aggregation of gene expression data from various cell types, yielding only a mean expression level. In our research, we aimed to accurately estimate cell-type proportions within actual bulk-tissue RNA-seq samples and identify death patterns among multiple neural cell subtypes in brain tissue, so we employed the deep learning-based Scaden algorithm [48]. This algorithm, specifically developed for deconvolution, is trained on simulated bulk-tissue RNA-seq data, which are derived from tissue-specific single-cell RNA-seq data [49]. We utilized brain-specific training data generated by simulating artificial bulk RNA-seq samples. Prior to training the Scaden model for 5000 steps, a measure to prevent overfitting was used, as recommended by the creators. We ensured gene and feature scale consistency within our datasets. Subsequently, cell-type proportion predictions were conducted via Scaden. The data obtained from this process will be instrumental in analysing cell types and constructing models to understand the patterns of neural cell death.

### Construction of a dynamic interactive model between cell death patterns and cell types

To construct a dynamic model elucidating the interactions between multiple neural cell types and various cell death modalities, we established four distinct phases. Initially, we generated five datasets encompassing core genes associated with apoptosis, autophagy, ferroptosis, necroptosis, and parthanatos. These gene sets were curated from Cell Snapshot (https://www.cell.com/snapshots) [50], which are considered by the current scientific community to be the most important regulatory routes in neurodegenerative diseases,serving as the death mode dataset for later analysis. In the second phase, we extracted the TPM values of these genes, forming the basis for subsequent interaction network construction. This extraction process resulted in a comprehensive gene expression matrix. The third phase involved integrating the cell death-related gene matrix with the matrix of neural cell subtypes. This integration was accomplished through a Mantel test [51]; the Mantel test was conducted via 1,000 permutations to ensure robustness, and the Pearson correlation coefficient was used as the distance measure. The resulting interactive signature model was further refined by incorporating Pearson correlation coefficients, Mantel test correlation values (R), and adjusted P values (p.adjust) calculated via the Benjamini‒Hochberg procedure to control for multiple comparisons. We defined a *P* value ≤ 0.05 as statistically significant, with a Mantel test *R* value > 0.5 indicative of a strong positive correlation. Finally, we quantified the interactions within the network to elucidate the dynamic interplay between neural cell types and cell death patterns throughout the progression of neurodegenerative diseases. This comprehensive approach helped elucidate the intricate relationships governing neuronal survival and death under these conditions.

### The correlation between nitric oxide synthase (NOS) and necroptosis

To investigate the correlation between NO and necroptosis, we performed correlation analysis of NOS1, NOS2, and NOS3, which are enzymes involved in NO production and may be the core genes involved in necroptotic cell death. The Pearson correlation coefficient was calculated via the ggcor R package.

### Mouse PD modelling

Healthy adult mice with body weights ranging from 20 to 25 g were randomly divided into the MPTP group, MPTP + Necrostatin-1s (Nec-1s) group, and saline group. All the animals were housed under standard conditions, including a constant temperature and humidity environment as well as a 12-h light‒dark cycle. The mice in the MPTP group received continuous intraperitoneal injections of MPTP (1-methyl-4-phenyl-1,2,3,6-tetrahydropyridine) starting from the first day of the experiment, with an injection dose of 20 mg/kg body weight. After the initial injection, subsequent injections were administered every 2 h for a total of four injections. The mice in the MPTP + Nec-1s group received Nec-1s intraperitoneal injections 3 days before MPTP administration, with the first dose being 10 mg/kg, followed by daily injections of 5 mg/kg until the behavioural experiments. The animals in the saline group received an equivalent volume of physiological saline, and on the 7th day, the success of the model was determined by tyrosine hydroxylase (TH) staining.

### Behavioural testing

On the 7th day after injection, behavioural testing was conducted on all surviving animals to assess the impact of MPTP injection on motor function. The tests included the rotarod test, which was used to evaluate the motor coordination and balance of the animals. The animals were placed on a rotating rod, and the time they were able to stay on the rod or until they fell off was recorded. The pole test was used to assess the movement disorders in mice. The animals were placed at the top of a vertical pole, and recorded the time taken for them to descend to the ground.

### TH staining of the substantia nigra

After the completion of the behavioural experiments, the animals were deeply anaesthetized to ensure painless euthanasia. The whole brain tissue was immediately removed and rinsed with physiological saline to remove blood and residual tissue. The brain tissue was fixed in a 4% paraformaldehyde solution and stored at 4°C for 24 h. After fixation, the brain tissue underwent stepwise dehydration, clarification, and paraffin embedding, followed by embedding in paraffin. The brain tissue was sectioned into 3.5 μm thick continuous slices via a microtome, and the slices were mounted on glass slides for immunohistochemical staining. After deparaffinization and antigen retrieval, the slices were stained with the dopaminergic neuron-specific antibody TH. The stained slices were observed under a light microscope, and the number of TH-positive cells was recorded and analysed. The potential protective effect of Nec-1s on MPTP-induced symptoms was assessed on the basis of the intensity of TH staining.

### Foetal mouse midbrain tissue isolation and primary neuron culture

Pregnant C57BL/6 mice at 14 days of gestation were euthanized swiftly with CO_2_ and placed on a prechilled dissection dish. Following rapid disinfection and uterus separation, the uterus was transferred to prechilled D-Hank's solution. With a stereomicroscope, foetal mouse midbrain tissue was meticulously dissected via microinstruments, and the soft meninges and vascular membranes were removed from the brain tissue. The dissected tissue was fully minced into a slurry on ice. The thoroughly minced midbrain tissue was then thoroughly mixed with 1 ml of 0.05% trypsin and incubated in a 37°C CO_2_ cell culture incubator for 15 min. The digestion process was halted by addition of Dulbecco's modified Eagle’s medium (DMEM) containing 10% foetal bovine serum and then by low-speed centrifugation (1000 rpm for 5 min), followed by washing and settling. The tissue was aspirated with DMEM, transferred to an appropriate volume of neurobasal medium supplemented with 2% B27, and repeatedly pipetted to generate a single-cell suspension. The final density was adjusted to 5 × 10^5^ cells/ml on the basis of the cell count results, and an appropriate amount of the cell suspension was seeded into a 6-well plate (coated with poly-L-lysine) and placed in a 37°C CO_2_ cell culture incubator. After 8 h, the culture medium was replaced entirely, and subsequently, every two days, half the neurobasal medium volume was replenished to maintain cell culture. The cells were ready for experimentation after 8 days [52].

### Trypan blue staining

A 4% stock solution of Trypan blue was prepared with Locke's buffer, and the solubilization process was facilitated by vertexing. Thereafter, the solution was subjected to filtration and stored at 4°C. Prior to staining, the stock solution was diluted with cell culture medium to a final concentration of 0.4% to create the working staining solution. The cells seeded in a 6-well plate were treated with the appropriate agents, after which the spent medium was removed. Then, 1 ml of 0.4% Trypan blue staining solution was added, and the cells were stained for 5 min. The staining outcome was subsequently immediately observed under a microscope and photographed.

### Neural cell line culture and drug treatment

The human neuroblastoma cell line SH-SY5Y was purchased from the China Typical Culture Collection Center of the School of Life Sciences, Wuhan University. The cells were cultured in high-glucose DMEM (12800, Gibco, USA) supplemented with 10% foetal bovine serum (A5669701, Gibco, USA), 100 units/ml penicillin and 100 μg/ml streptomycin (15070063, Gibco, USA) at 37°C, 5% CO_2_ and 95% humidity. We established a PD cellular model by treating SH-SY5Y cells with 100 nmol/l rotenone (R8875; Sigma‒Aldrich, USA) for 24 h [53], which has been reported to effectively simulate neuronal damage in PD [53–56]. For analysis of the role of NO in the mechanism of neurodegeneration, the exogenous NO donor GSNO (57564–91-7, Sigma, USA) was applied to SH-SY5Y cells at a stimulating concentration of 500 μmol/l, either alone or in combination with rotenone, to elicit specific cellular effects. For the primary neuron assay, 100 µM GSNO was used to treat midbrain neurons derived from mice to elucidate the role of NO-induced necroptosis.

### Determination of cell death patterns in PD cellular models

We treated neural cells with specific pharmacological inhibitors, including an apoptosis inhibitor [Z-VAD-FMK (Z-VAD), 219007, Sigma, USA] at a stimulating concentration of 2.5 mmol/l; an autophagy inhibitor [3-methyladenine (3-MA), SAE0107, Sigma, USA] at a stimulating concentration of 5 μmol/l; a ferroptosis inhibitor [ferrostatin-1 (Fer-1), 347174–05-4, Sigma, USA] at a stimulating concentration of 100 μmol/l; a necroptosis inhibitor (Nec-1s, 852391–15-2, Sigma, USA) at a stimulating concentration of 50 μmol/l; and a parthanatos inhibitor [olaparib (Ola), 763113–22-0, Sigma, USA] at a stimulating concentration of 10 μmol/l, to validate the cellular damage pathways of apoptosis, autophagy, ferroptosis, necroptosis, and parthanatos.

### MTT assays to evaluate cell viability

To determine cell viability, we conducted an MTT (3580GR001, BioFroxx, China) assay on each group. The cells were subsequently centrifuged at 1,000 rpm for 5 min and seeded in a 96-well plate at a density of 1 × 10^5^ cells per well with 200 μl of culture medium. Twenty-four hours after drug treatment, 20 μl of MTT solution (0.5 g/l) was added to each well, followed by a 3-h incubation. The supernatant was then removed, and 150 μl of DMSO was added. The plate was shaken at 130 rpm for 5 min. The absorbance was measured at 565 nm.

### Mitochondrial membrane potential (MMP) assay and nuclear staining

To assess the energy charge status of the cell, we conducted MMP measurements. The cells in each group were washed twice with Locke's buffer after stimulation. Then, 1 ml of MitoTracker™ Red CMXRos dye (M7512, Invitrogen, USA) was added to each group, and the cells were incubated at 37°C for 30 min. After the dye was removed, the cells were stained with Hoechst 33342 (CB2261, Sigma, USA) diluted in liquid culture medium, followed by incubation in the dark for 10 min. The staining solution was then discarded, and the cells were fixed with prechilled (-20°C) methanol for 30 min. After the methanol was discarded, each group was washed with 1 ml of Locke's solution. The cells were subsequently observed under a fluorescence microscope, and images were captured for further analysis.

### Western blot analysis

Protein lysates were prepared by lysing the cells in each well of a 6-well plate with 200 μl of RIPA buffer. The protein concentration was determined via the BCA method. The lysates were subsequently mixed with 5 × SDS loading buffer and boiled for later use. The samples were then loaded into separate lanes for SDS‒PAGE, which was initially run through the stacking gel at 80 V and then through the separating gel at 120 V. After electrophoresis, the proteins were transferred to a PVDF membrane, which was then blocked with 5% skim milk. The membranes were incubated overnight at 4°C with specific primary antibodies: anti-caspase-3 (dilution 1:1000, ab32351, Abcam, UK), anti-cleaved caspase-3 (1:1000, ab32351, Abcam, UK), anti-RIP1, anti-phospho-RIPK1, anti-RIP3, anti-phospho-RIP3, anti-phospho-MLKL, anti-MLKL, and anti-beta-actin antibodies included in the Necroptosis Antibody Sampler Kit (#98110, Cell Signaling Technology, USA). Later, the membrane was washed three times with TBST and incubated with an HRP-linked secondary anti-rabbit antibody (dilution 1:5000, ab288151, Abcam, UK) for 1 h. After three further TBST washes, the membrane was developed with an enhanced chemiluminescence (ECL) substrate, and the protein bands were visualized. Semiquantitative analysis of the bands was performed via ImageJ.

### Statistical analysis

All the statistical analyses were performed via R and SPSS 24.0. The means ± SDs were used to describe continuous variables that were normally distributed. *P* < 0.05 indicated statistical significance. To evaluate the performance of the necroptosis molecular diagnostic test and its efficacy for a disease stratification biomarker across PD, AD, and ALS, we conducted multifactorial logistic regression analyses. The receiver operating characteristic (ROC) curve was subsequently plotted to calculate the area under the curve (AUC), sensitivity, and specificity.

## Supplementary Information

Supplementary Material 1.

Supplementary Material 2.

Supplementary Material 3.

## Data Availability

The datasets generated and analysed during the current study are available in GSE216281, GSE203206, GSE219278, GSE168496, GSE263588, GSE252291 and GSE260487 (https://www.ncbi.nlm.nih.gov/geo/).
