# Supplementary material for: Identification of nitric oxide-mediated necroptosis as the predominant death route in Parkinson’s disease
Source: Mol Biomed. 2024 Oct 24;5:44. doi: 10.1186/s43556-024-00213-y (PMC11499487; doi:10.1186/s43556-024-00213-y)
Supplement: Supplementary file 1 — Supplementary Material 1. [file 43556_2024_213_MOESM1_ESM.docx]

**Identification of nitric oxide-mediated** **necroptosis as the predominant death route in Parkinson’s disease**

Ting Zhang^#1,2^, Wenjing Rui^#3^, Yue Sun^#1,2,4^, Yunyun Tian^1,2^, Qiaoyan Li^1,2^, Qian Zhang^1,2^, Yanchun Zhao^1,2^, Zongzhi Liu^*3,5^, Tiepeng Wang^*1,2,6^

*1. School of Medicine, Shihezi University, Shihezi, 832000, China.*

*2. Key Laboratory of Xinjiang Endemic and Ethnic Diseases, School of Medicine, Shihezi University, Shihezi, 832000, China.*

*3. Changping Laboratory, Beijing, 102206. China.*

*4. Prenatal diagnosis center of Urumqi Maternal and Child Health Hospital, Urumuqi, Xinjiang 830000, China*

*5. Key Laboratory of Genomic and Precision Medicine, Beijing Institute of Genomics, Chinese Academy of Sciences, Beijing, 100101, China.*

*6. Key Laboratory of Biomacromolecules (CAS), National Laboratory of Biomacromolecules, CAS Center for Excellence in Biomacromolecules, Institute of Biophysics, Chinese Academy of Sciences, Beijing 100101, China.*

**Key words:** Nitric oxide, Necroptosis，Parkinson's disease, Neurodegenerative disease

**Runing Title:** Nitric Oxide-Mediated Necroptosis in Parkinson's Disease

**^#^ Ting Zhang, Wenjing Rui, Yue Sun contribute equally.**

**^*^ Correspondence:**

Zongzhi Liu

Email: lzz871215@163.com

Tiepeng Wang

Email:[wangtiepeng2002@163.com](mailto:wangtiepeng2002@163.com)


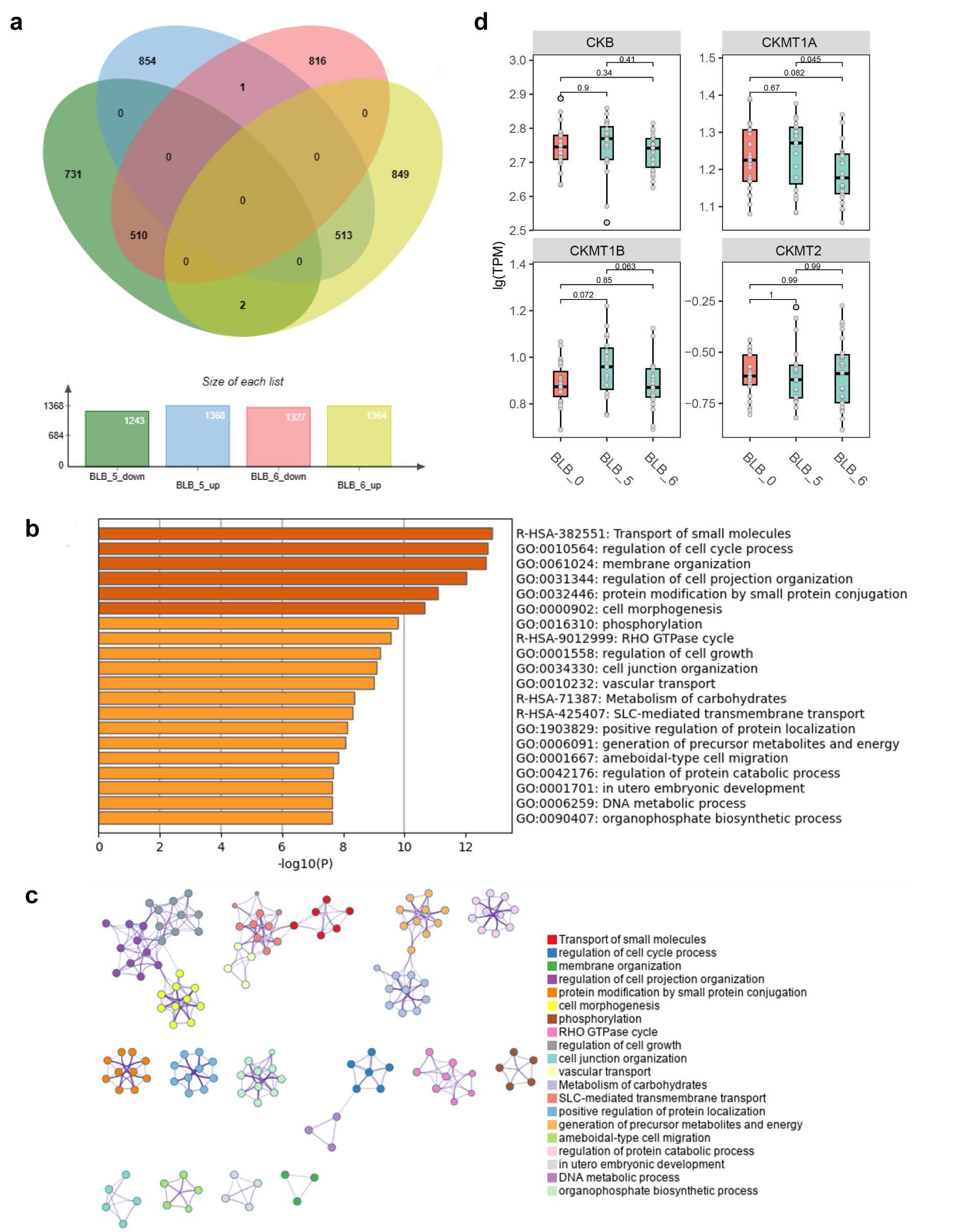


**Figure S1 Statistics of DEG** **at different pathological stages.**

(a) Venn diagram exhibited the DEGs at different pathological stages.

(b) Pathway enrichment of DEGs between BLB5 and BLB0.

(c) Pathway interaction network between BLB5 and BLB0.

(d) Expression of creatine kinase family genes at different pathological stages (The x-axis represents different periods, the y-axis represents lgTPM, and p<0.05 indicates a statistically significant difference).


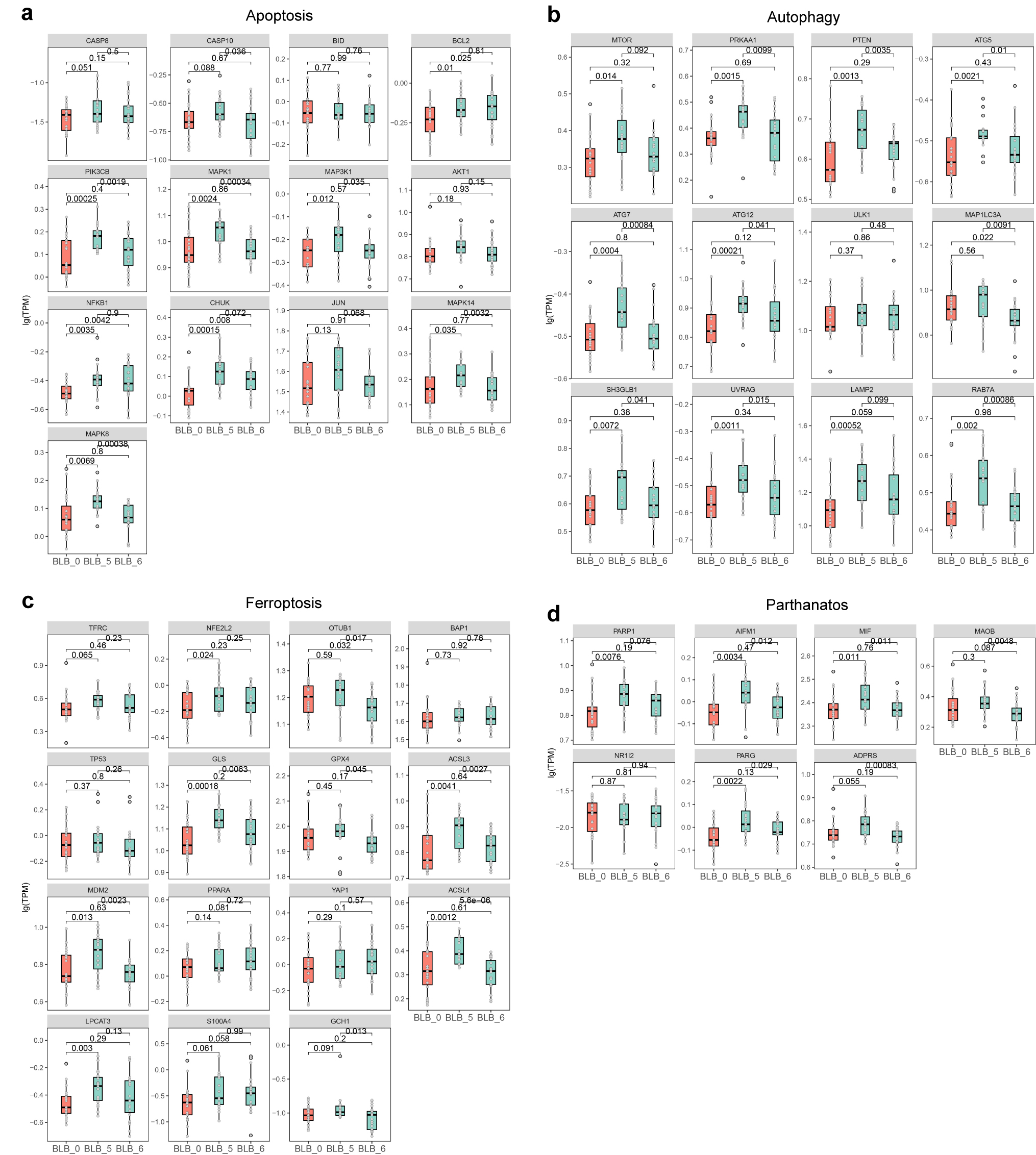


**Figure S2** **Core gene expression at different pathological stages.**

(a~d) Represent the pathological stage-dependent expression of key regulatory genes in apoptosis, autophagy, ferroptosis, and parthanatos pathways respectively in PD samples.


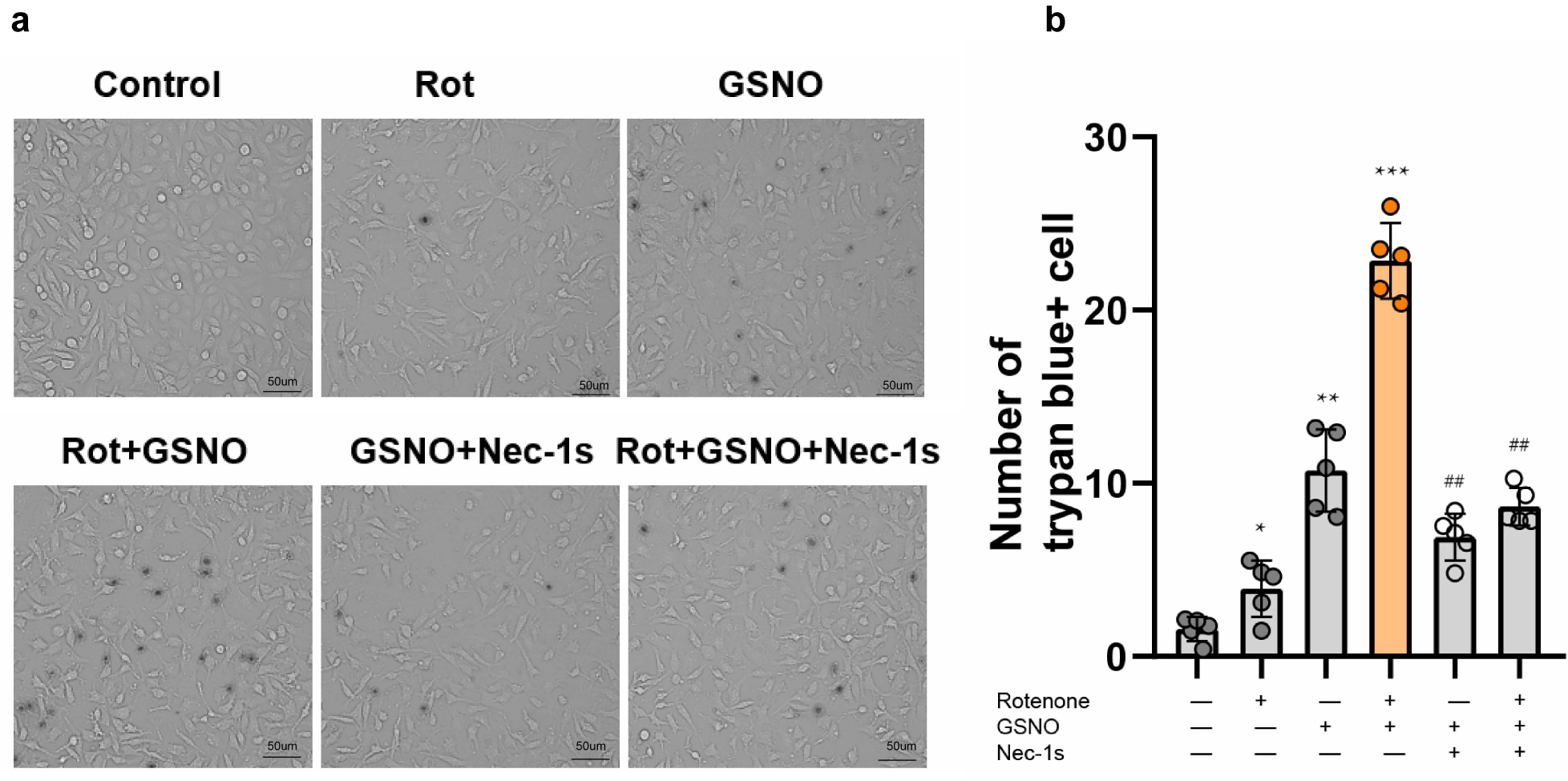


**Figure S3 Nitric oxide compromised the integrity of cell membrane in necroptotic cells.**

(a)The SH-SY5Y cell line was stimulated with rotenone alone or in combination with the nitric oxide donor GSNO, with or without the necroptosis inhibitor Nec-1s, for a period of 8 hours prior to staining with trypan blue and subsequent photography.

(b)Statistics of necroptosis. Ten random fields of view were selected, and the proportion of stained cells within each field was used to characterize the extent of necroptotic cell death (*, compared with control group, **, P<0.01, ***, P<0.001.#, compared with rotenone+ GSNO group, ##, P<0.01.)


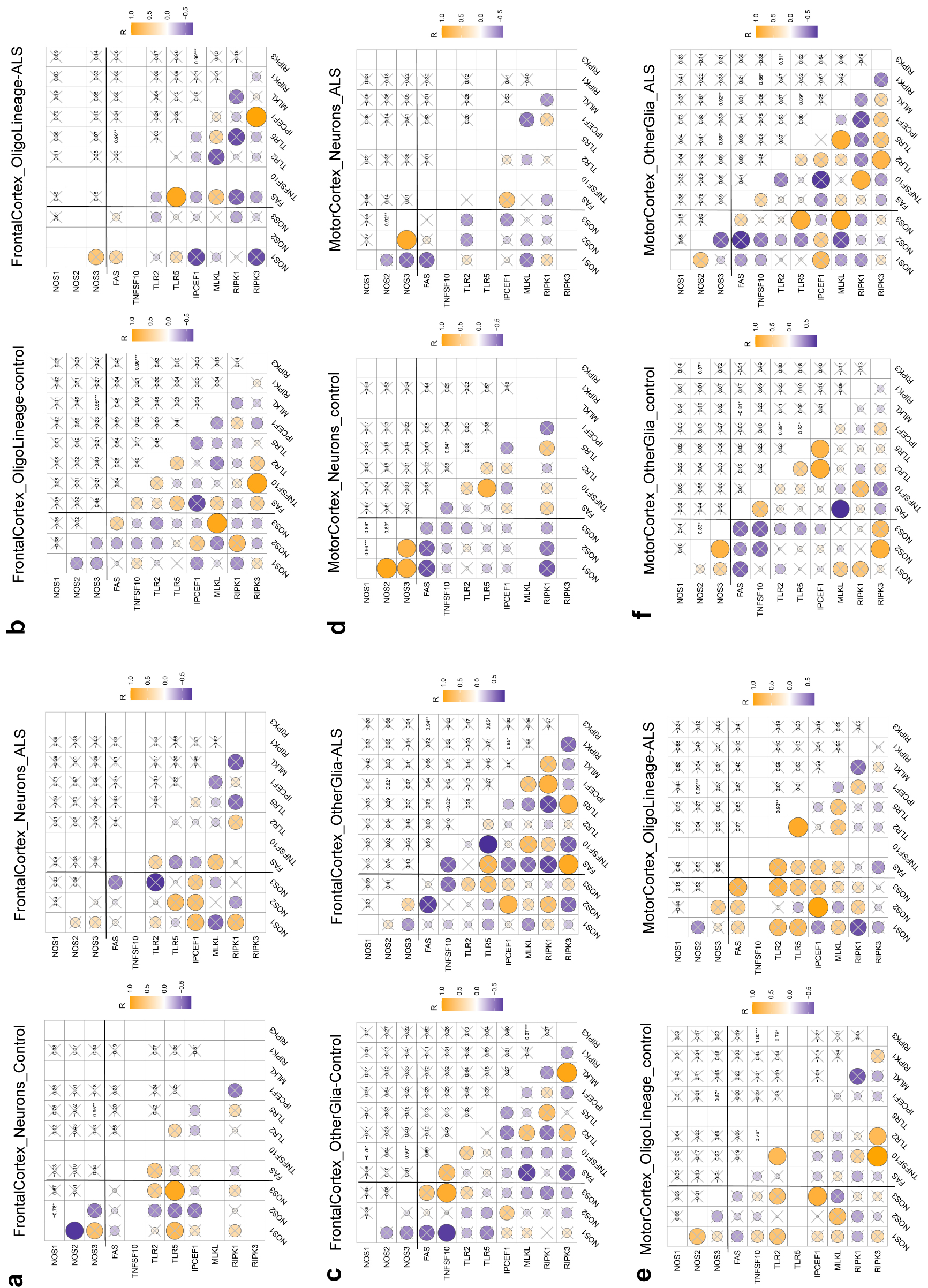


**Figure S4 The pervasiveness of NO-mediated necroptosis across various brain regions and among diverse cell types in individuals with ALS.**

Figures A to F depict the correlations between genes of the NOS family and those involved in necroptosis within distinct neural cell populations: (a) frontal cortex neurons, (b) frontal cortex oligodendrocyte lineage, (c) other glial cells of the frontal cortex, (d) motor cortex neurons, (e) oligodendrocyte lineage of the motor cortex, and (f) other glial cells of the motor cortex.
