# Supplementary material for: Identification of nitric oxide-mediated necroptosis as the predominant death route in Parkinson’s disease
Source: Mol Biomed. 2024 Oct 24;5:44. doi: 10.1186/s43556-024-00213-y (PMC11499487; doi:10.1186/s43556-024-00213-y)
Supplement: Supplementary file 3 — Supplementary Material 3. [file 43556_2024_213_MOESM3_ESM.pptx]

## Slide 1
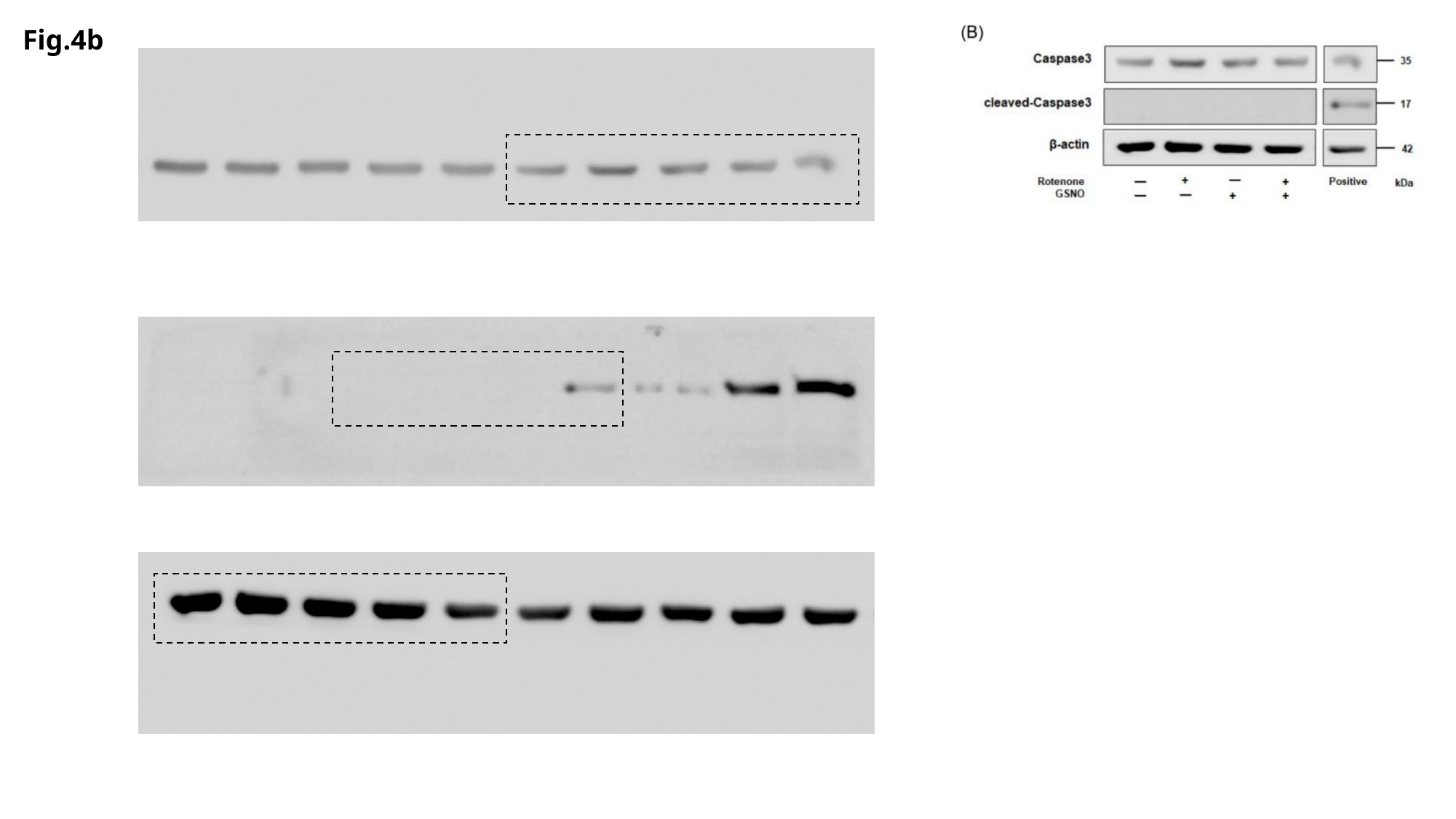

Fig.4b

## Slide 2
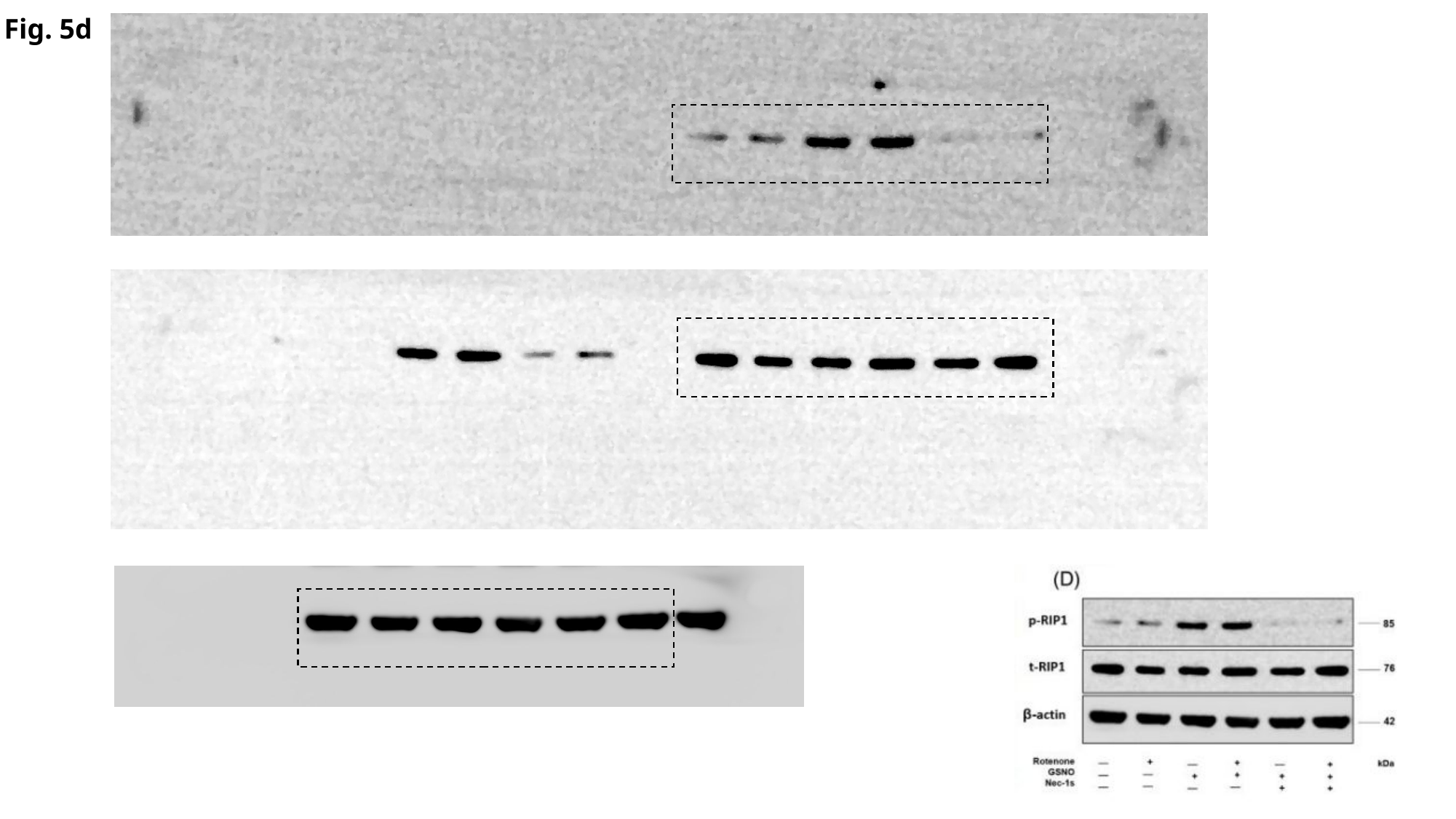

Fig. 5d

## Slide 3
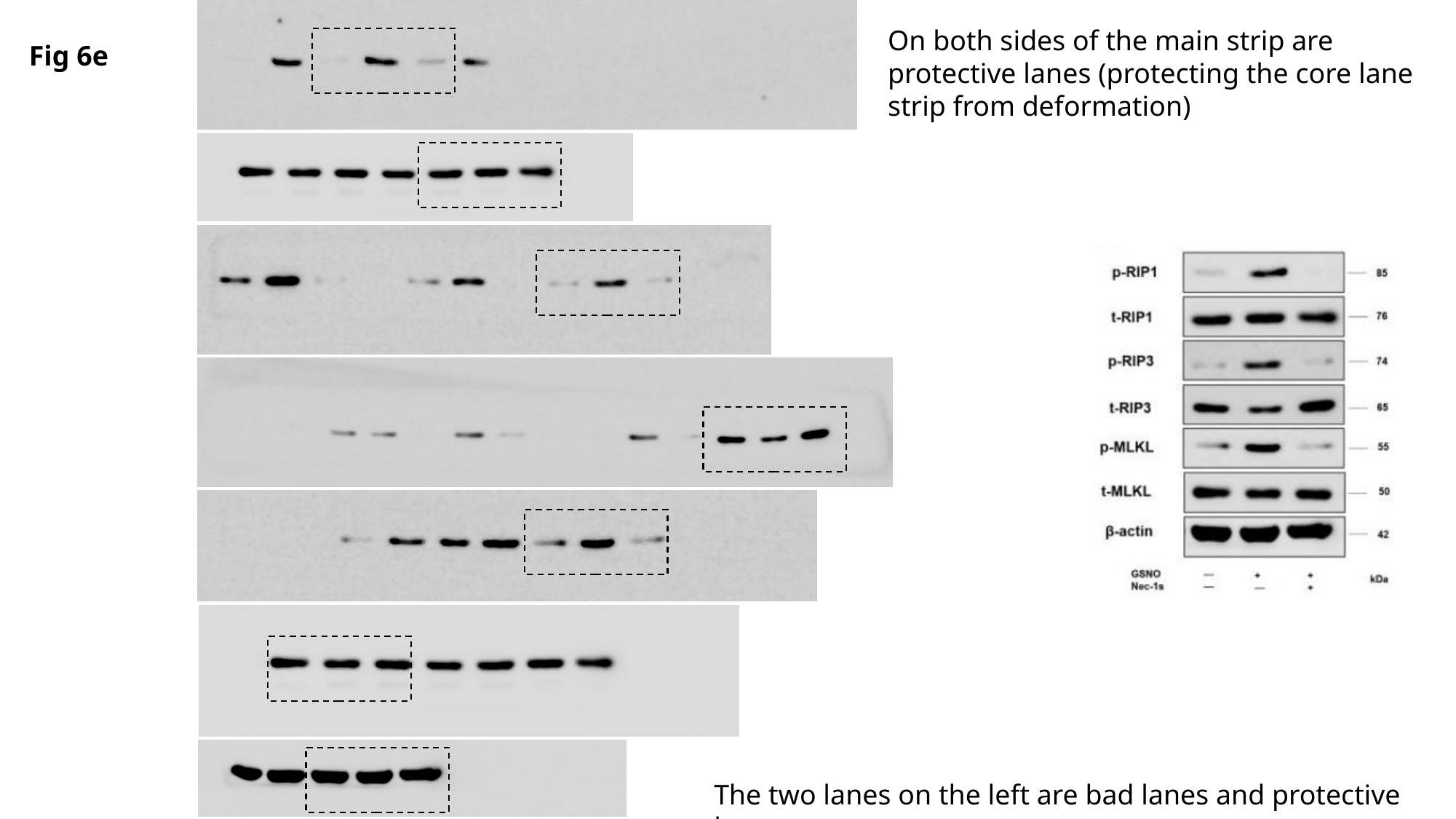

On both sides of the main strip are protective lanes (protecting the core lane strip from deformation)
Fig 6e
The two lanes on the left are bad lanes and protective lanes

## Slide 4
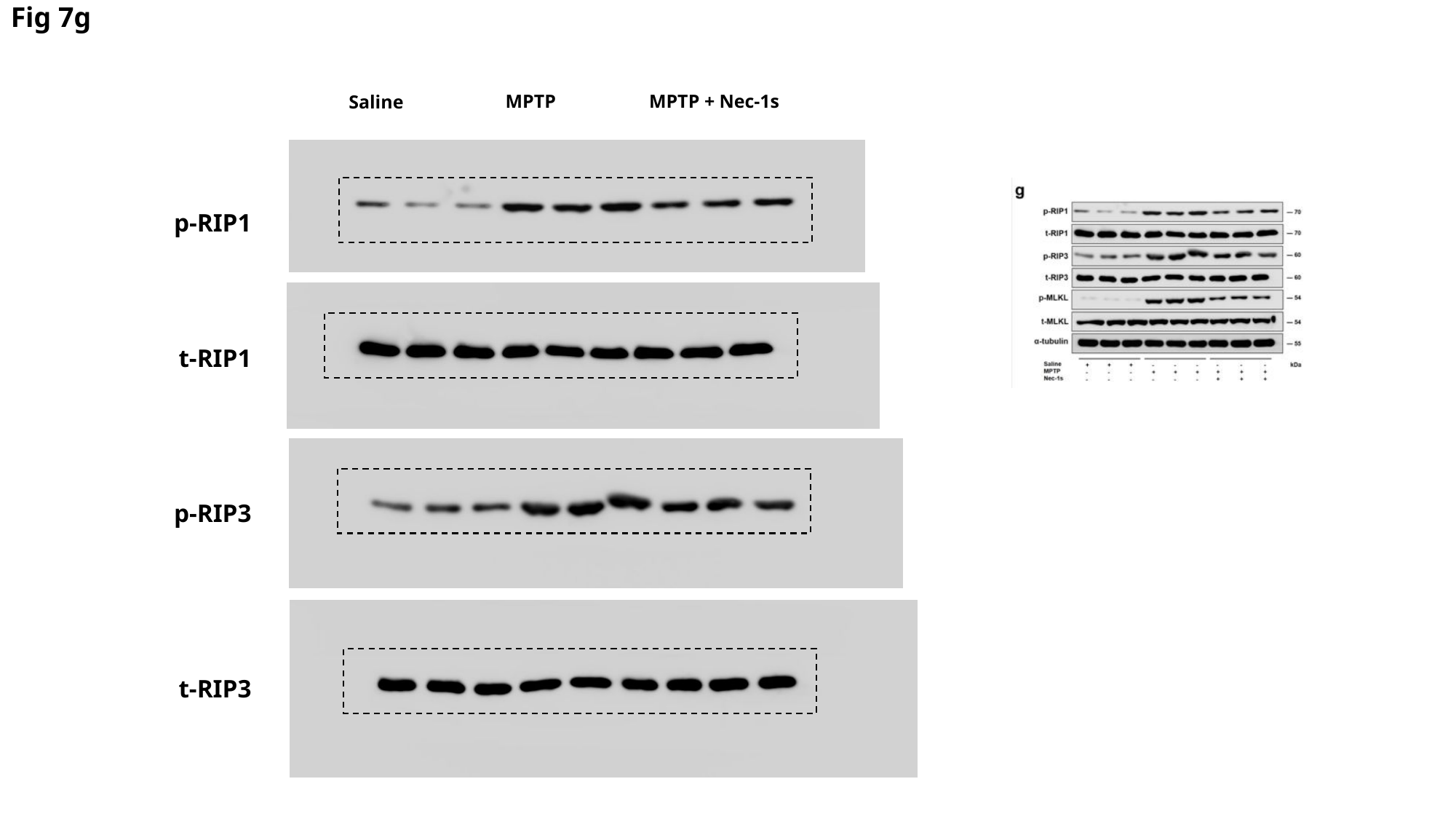

Fig 7g
MPTP
MPTP + Nec-1s
Saline
p-RIP1
t-RIP1
p-RIP3
t-RIP3

## Slide 5
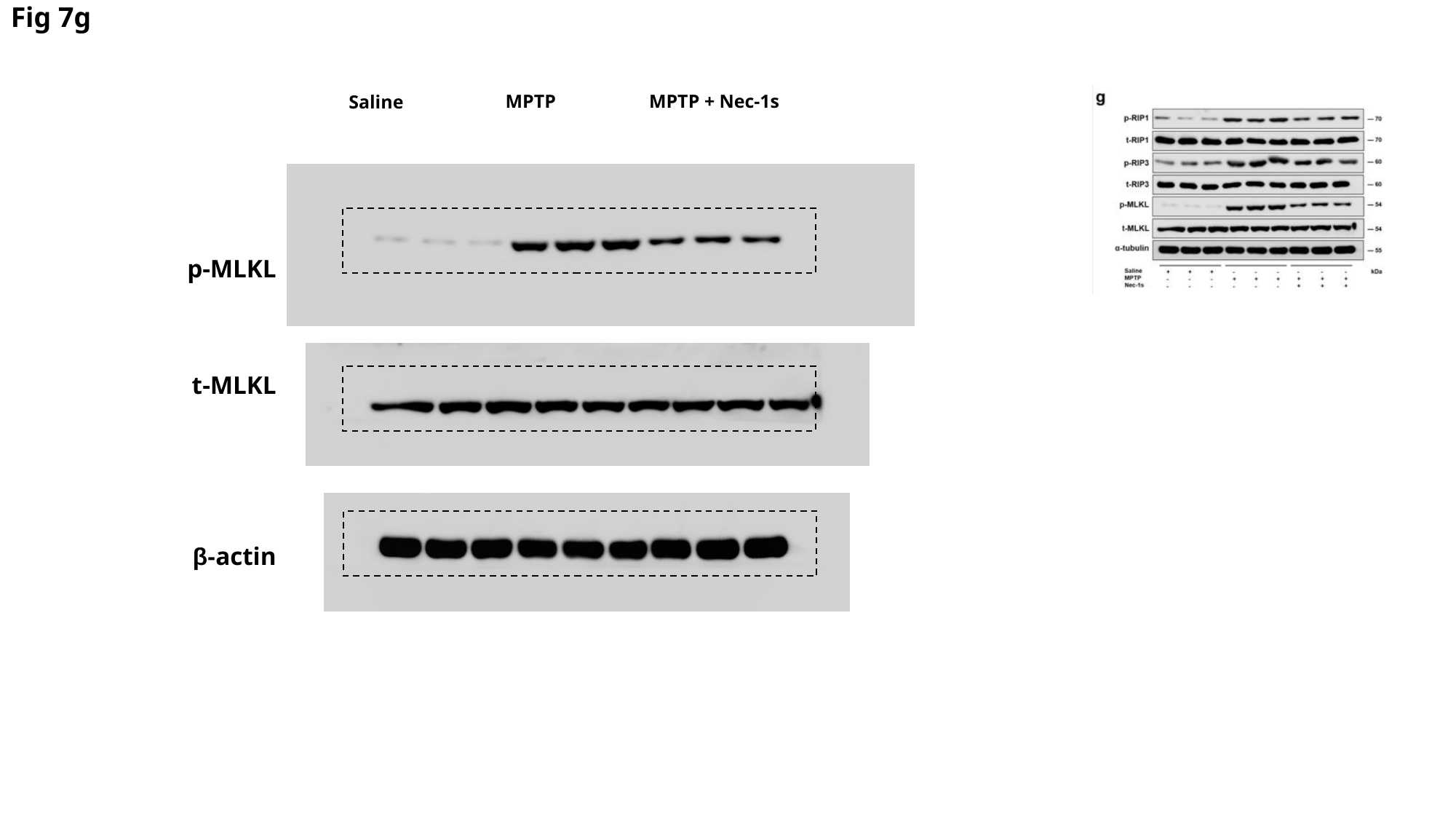

Fig 7g
MPTP
MPTP + Nec-1s
Saline
p-MLKL
t-MLKL
β-actin
